# Universal roles of hydrogen in electrochemical performance of graphene: high rate capacity and atomistic origins

**DOI:** 10.1038/srep16190

**Published:** 2015-11-05

**Authors:** Jianchao Ye, Mitchell T. Ong, Tae Wook Heo, Patrick G. Campbell, Marcus A. Worsley, Yuanyue Liu, Swanee J. Shin, Supakit Charnvanichborikarn, Manyalibo J. Matthews, Michael Bagge-Hansen, Jonathan R.I. Lee, Brandon C. Wood, Y. Morris Wang

**Affiliations:** 1 Physical and Life Sciences Directorate, Lawrence Livermore National Laboratory, Livermore, California 94550, USA

## Abstract

Atomic hydrogen exists ubiquitously in graphene materials made by chemical methods. Yet determining the effect of hydrogen on the electrochemical performance of graphene remains a significant challenge. Here we report the experimental observations of high rate capacity in hydrogen-treated 3-dimensional (3D) graphene nanofoam electrodes for lithium ion batteries. Structural and electronic characterization suggests that defect sites and hydrogen play synergistic roles in disrupting *sp*^*2*^ graphene to facilitate fast lithium transport and reversible surface binding, as evidenced by the fast charge-transfer kinetics and increased capacitive contribution in hydrogen-treated 3D graphene. In concert with experiments, multiscale calculations reveal that defect complexes in graphene are prerequisite for low-temperature hydrogenation, and that the hydrogenation of defective or functionalized sites at strained domain boundaries plays a beneficial role in improving rate capacity by opening gaps to facilitate easier Li penetration. Additional reversible capacity is provided by enhanced lithium binding near hydrogen-terminated edge sites. These findings provide qualitative insights in helping the design of graphene-based materials for high-power electrodes.

The commercial applications of graphene materials for energy storage devices, including lithium ion batteries (LIBs) and supercapacitors^1^,^2^,^3^,^4^,^5^,^6^, hinge critically on our ability to produce these materials in large quantities and at low-cost^1^,^7^. The wet chemistry approach (i.e., liquid phase exfoliation and reduction of graphene oxide)^8^,^9^ can produce graphene in tons and holds much promise. However, impurities—especially atomic hydrogen — exist ubiquitously in graphene materials made by chemical methods, and their effect on lithium storage capability remains little understood despite some rather important roles that these cross contaminants could play^10^. Theory and experiments have suggested that hydrogen adsorbents impact the electronic structures of graphene^11^,^12^,^13^ and are expected to play a significant role in influencing the lithium storage capacity when applied as anodes in LIBs. Nonetheless, the conclusive evidence for this behaviour has not been forthcoming.

Earlier studies on various carbon materials (e.g., most are soft carbon from pyrolysis of organic precursors) generally pointed at an increased lithium storage capacity with increasing hydrogen content^14^. The exact mechanisms underpinning this empirically observed behaviour remain a subject of ongoing debate and it is unclear whether this phenomenon also occurs for graphene. A main challenge has been to control the hydrogen content and location in graphene materials, a subject that is also of great interest to hydrogen storage applications^15^,^16^. Control experiments on graphene single sheets, on the other hand, indicate that the hydrogenation of graphene could convert highly conductive zero-overlap semimetal graphene into an insulator (graphane)^11^. If this occurs, hydrogen may adversely impact lithium storage due to the loss of electrical conductivity. A brief survey of the literature on graphene nanosheets made by wet chemistry and thermal exfoliation methods fails to yield any clear-cut correlations among synthetic methods, hydrogen content, and lithium storage capacities^17^, suggesting the rather complex roles of impurities and defect structures in influencing lithium storage behaviour of graphene materials^18^. Computer simulations in fact showed that perfect graphene lacks lithium storage mechanisms^19^, and that defect structures are prerequisite for lithium storage^20^,^21^. In practice, however, defect sites of graphene tend to bind functional groups that often contain hydrogen (e.g., hydroxyl, carboxyl, amine, hydrogen). This underscores the universal significance of understanding the roles of hydrogen in influencing the electrochemical behaviour of graphene.

To investigate the involvement of hydrogen and hydrogenated defects in the lithium storage ability of graphene, we apply various heat treatment conditions combined with hydrogen exposure and interrogate the electrochemical performance of 3D graphene nanofoam (GNF) electrodes. Our studies use self-assembled 3D graphene nanofoams due to their numerous potential applications, including hydrogen storage, catalysis, filtration, insulation, energy sorbents, capacitive desalination, supercapacitors, and LIBs^22^. The binder-free nature of graphene 3D foam makes them ideal for mechanistic studies without the complications caused by additives. We report a drastically improved rate capacity in GNFs after hydrogen treatment, which we attribute to complex interactions between defects and dissociated hydrogen that alter the chemistry and morphology of the substrate. We discuss the atomistic origins of this behaviour through a series of control experiments and multiscale simulations, and suggest its use as a strategy for optimizing lithium transport and reversible storage in graphene-based anode materials.

## Results

### Structural and electronic characteristics

**Figure 1:**
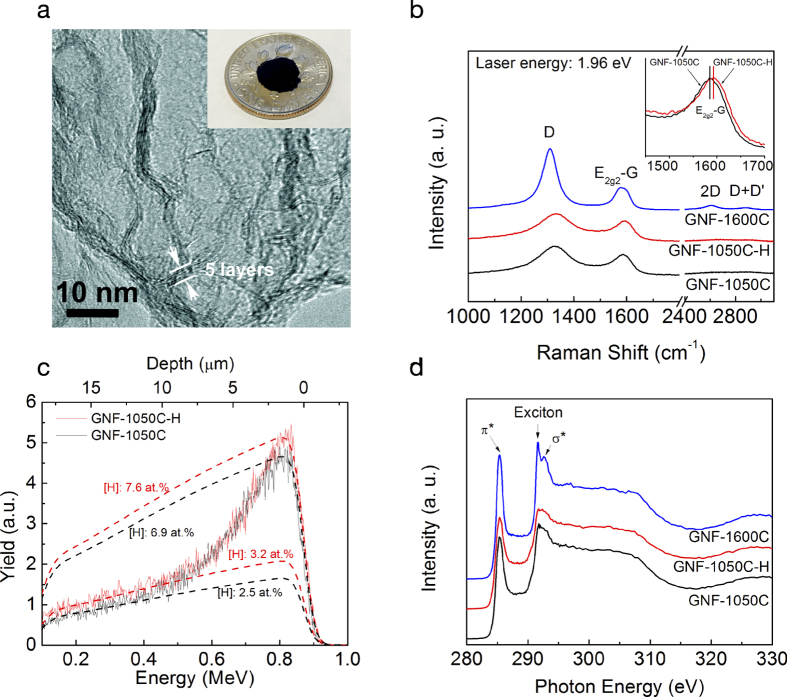
Structural and electronic structure of 3D graphene nanofoams (GNFs). (**a**) Transmission electron micrograph (TEM) of GNF-1050C sample before H_2_ treatment. The inset is an optical image of GNF disk sitting on top of a US penny. (**b**) Raman spectra of three representative GNFs after various high temperature and/or H_2_ treatment conditions. The inset is the zoomed-in Raman spectra of two comparison samples (i.e., GNF-1050C vs. GNF-1050C-H). A blue shift of G band is observed after H_2_ treatment. (**c**) Elastic recoil detection analysis (ERDA) of hydrogen content in GNF-1050C and GNF-1050C-H. An average H content of 3.6 at.% and 4.3 at.% is revealed before and after H_2_ treatment. (**d**) X-ray absorption spectra (XAS) of three GNF samples (see text for detailed discussion).

Our electrode materials are 3D GNFs (250  μm thick, 5–10 mm in diameter, inset of Fig. 1a) constructed from graphene oxide sheets through a sol-gel process (Methods)^22^. A series of comparison GNF samples are investigated, namely, GNF-1050C, GNF-1050C-H, GNF-1050C-H2, GNF-1600C, GNF-1600C-H, GNF-2000C, and GNF-2500C. The processing conditions of each sample are shown in Table 1. Due to a large number of GNF samples involved in our studies, the data presented in this work mainly focus on three key samples that help to yield the most valuable information for computational understanding. These samples are GNF-1050C, GNF-1050C-H, and GNF-1600C. Note that samples with ‘–H’ labels are those treated in H_2_ environment. The original 3D structure of these three GNFs can be seen in Fig. S1 of Supplementary Information (SI). The transmission electron micrograph (TEM) of the starting material GNF-1050C in Fig. 1a indicates that it consists of relatively transparent regions attributed to few-layer graphene and dark rippled areas, where occasionally stacked layers are visible. Most interlayers are observed to be twisted due to the strain that is geometrically necessary to construct 3D GNFs. The relatively rough features on the transparent graphene layers suggest the defective nature of these entities. Raman spectroscopic analysis in Fig. 1b reveals that at the laser excitation energy of 1.96 eV, GNF-1050C has a D/G band intensity ratio of 1.45 ± 0.03, consistent with the defective nature of graphene. After annealing in a hydrogen environment (4.0 at.% H_2_ + Ar) at 400 °C (Methods), we note that the D/G band intensity ratio of graphene (GNF-1050C-H) is marginally changed (1.48 ± 0.03). However, a blue-shift of E_2g2_ mode (i.e., G-band) is observed in GNF-1050C-H (inset of Fig. 1b)^23^, suggestive of additional contribution from non-zone centre phonons, likely due to disorder at domain boundaries. The energy dispersive double resonant D-band at different laser energies (SI, Fig. S2) exhibits a slope of ~60 cm^−1^/eV for GNF-1050C and GNF-1050-H that is larger than the typical value for graphene (~38 cm^–1^/eV)^24^. The variation in D-band frequency obtained with the same laser energy may be ascribed to a slight variation in force-constants between these two materials, with the GNF-1050C-H exhibiting tighter binding^25^.

The D/G band intensity ratio increases to 2.67 ± 0.18 when GNF is annealed at a higher temperature of 1600 °C in Ar environment (GNF-1600C). The increased D/G band ratio at higher annealing temperatures is counterintuitive but suggests that the GNF-1050C and the GNF-1600C belong to Stage II and Stage I defect region materials with an estimated graphene domain size of 1.4 nm and 10.4 nm^18^,^26^,^27^ (SI, Fig. S3), respectively (Methods). The Brunauer-Emmett-Teller (BET) surface area measurement and porosimetry characterization (SI, Fig. S4) reveal that H_2_-treatment leads to a slight shift towards smaller pores and a marginal decrease of surface area, so does the higher temperature treatment (Table 2). Elastic recoil detection analysis (ERDA) in Fig. 1c indicates that the hydrogen distribution inside GNFs is generally nonuniform with higher hydrogen on the surface and levels off after ~10 μm deep. The average H-content is found to increase from 3.6 at.% in GNF-1050C to 4.3 at.% in GNF-1050C-H (Methods); i.e., H_2_ treatment increases the total atomic hydrogen level in GNFs. Interestingly, we do not observe an obvious increase of d-spacing for hydrogen treated sample (SI, Fig. S5).

The electronic structure of three GNF samples (GNF-1050C, GNF-1050-H, GNF-1060C) is investigated by carbon *K*-edge X-ray adsorption spectroscopy (XAS), Fig. 1d. These spectra display features characteristic of *sp*^2^ carbon materials, including a sharp resonance at ~285.4 eV that is attributed to C(1s) → (C-C) π* transitions, a feature at ~291.5 eV arising from a core-hole (σ) exciton state^28^, and a manifold of resonances at higher energies primarily associated with C(1s) → (C-C) σ* transitions^29^. Subtle changes in the x-ray absorbance are observed following exposure of the GNF-1050C to 4.0 at.%-H_2_ environment at 400 °C. The GNF-1050C-H spectrum exhibits a slight reduction in intensity of both the C(1s) → (C-C) π* resonance and the C(1s) → (C-C) σ* resonance at ~292.5 eV with respect to the GNF-1050C data. In contrast, any changes in the intensity of the C(1s) → (C-H) σ* resonance fall within experimental error. The diminished intensity of the C-C π* and σ* resonances suggests a reduction in the overall *sp*^2^ character of the GNF^29^ following exposure to H_2_. In light of this assignment, the absence of any accompanying intensity changes in the C(1s) → (C-H) σ* is important because it eliminates possible mechanisms by which the proportion of *sp*^2^ carbon could decrease. In particular, the addition of molecular hydrogen across two adjacent *sp*^2^-hybridized carbon atoms (i.e. to yield two *sp*^3^-hybridized carbons) is omitted as a viable mechanism for the reduction in *sp*^2^ character because it would necessitate the observation of a corresponding increase in intensity of the C(1s) → (C-H) σ* resonance at ~ 289.5 eV^29^. For the GNF-1600C sample, we observe an increase in intensity of the C(1s) → (C-C) π* resonance, indicative of an increase in the proportion of *sp*^2^ hybridized carbon within the GNFs. This assignment is further supported by the observed sharpening and intensity increase in the core-hole exciton feature upon hydrogenation. Both features are characteristic of an increase in domain size and crystallinity of the GNF-1600C sample, consistent with Raman signatures.

### Electrochemical behaviour at various charge/discharge rates

**Figure 2:**
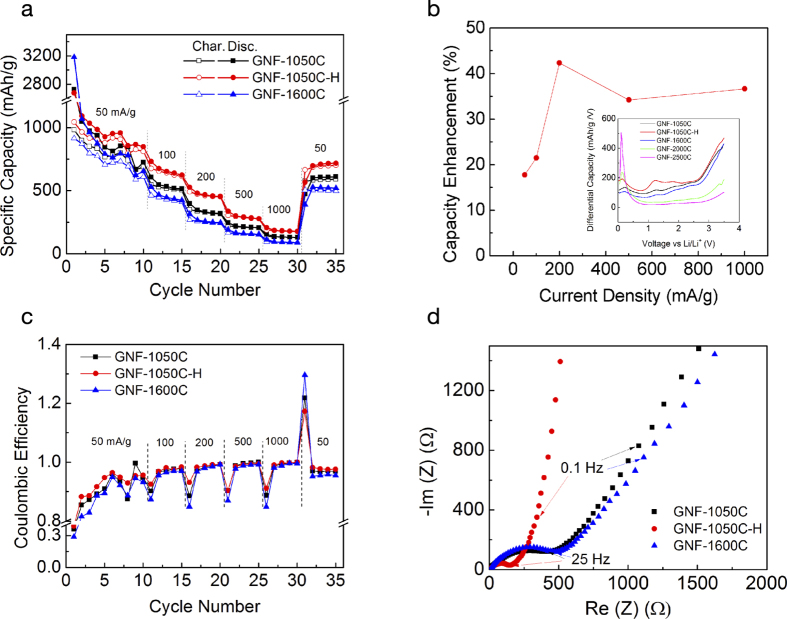
Electrochemical characteristics of 3D graphene nanofoams (GNFs). (**a**) Charge/discharge rate jump experiments show the improved rate performance after H_2_ treatment. (**b**) The percentage capacity enhancement at different charge/discharge rates before and after H_2_ treatment. The inset is the anodic differential capacity curves at various current densities at fifth cycle. (**c**) Coulombic efficiency of three representative GNF samples. Note that enhancement of Coulombic efficiency after H_2_ treatment. (**d**) Nyquist plots in impedance measurement imply easier charge transfer after H_2_ treatment.

The specific capacities obtained at different charge/discharge rates for the three GNF samples shown in Fig. 2a indicate that H_2_-treated sample (i.e., GNF-1050C-H) has substantially better rate capacity compared to the GNF-1050C sample, whereas the GNF-1600C has the worst rate performance (the voltage profiles of three samples at the 1^st^ and 5^th^ cycle can be seen in SI, Fig. S6). In addition, the intrinsically high capacity of GNF-1050C-H is manifested at 50 mA/g current density after 30 cycles. Since GNF-1050C-H and GNF-1050C have marginal different in terms of pore-size distribution and pore volume (Table 2), the significantly higher capacity and better rate performance in GNF-1050C-H cannot be simply attributed to pore size effects. We define the capacity enhancement as δ=(C_GNF-1050C-H_-C_GNF-1050C_)/C_GNF-1050C_ × 100%, where C_GNF-1050C-H_ and C_GNF-1050C_ are the delithiation capacities of H_2_-treated and reference GNF-1050C graphene samples, respectively. In Fig. 2b, we observe a δ value that varies with the charge rate and ranges from 17–43% after H_2_ treatment. At lower rates, δ increases sharply before generally saturating at higher rates. The maximum enhancement of ~43% is observed at the charge/discharge rate of 200 mA/g. This impressive improvement of rate capacity in H_2_-treated sample is surprising, given the fact that the overall hydrogen content in GNF-1050C-H was only slightly increased (Fig. 1c), whereas the oxygen content remains identical (~2.0 at.%) (SI, Fig. S7) as determined by Rutherford backscattering spectroscopy (RBS). This strong electrochemical performance enhancement is highly reproducible and is also observed in another set of control samples that were annealed in 100% H_2_ environment (SI, Fig. S8). The differential capacity curves of a series of GNF samples in the inset of Fig. 2b reveal two informative trends: (1) the lithium intercalation peaks (<0.5 V) shift towards lower potentials after H_2_ treatment (i.e., compare GNF-1050C-H with GNF-1050C), suggestive of easier intercalation processes; (2) higher temperature annealing without H_2_ leads to higher graphitization of graphene and thus stronger lithium intercalation peaks. However, the same higher temperature annealing also leads to the disappearance of multiple lithium reaction peaks in the voltage range of 1–3 V, causing low capacity in higher temperature annealed graphene materials (i.e., GNF-2000C and GNF-2500C, SI, Fig. S9). We observe that GNF-1600C has the largest initial lithiation capacity (~3182 mAh/g in Fig. 2a), but the lowest Coulombic efficiency (~28.7%), Fig. 2c. In comparison, the H_2_-treated GNF-1050C-H graphene shows a lower initial lithiation capacity (~2677 mAh/g), but the highest first-cycle Coulombic efficiency (~39.0%). The observation of a lower Coulombic efficiency in the less-defective GNF-1600C (see Fig. S3) compared to the GNF-1050C contrasts with the popular belief that surface functional groups on graphene (especially the oxygen-containing groups) tend to cause side reactions of electrolyte and thus lower Coulombic efficiencies^30^. Our RBS measurements in fact show much less oxygen content in GNF-1600C (Fig. S7). These results suggest that defective structures on graphene surface provide an overall benefit for achieving higher Coulombic efficiency despite their strong affinity to functional groups that are often cited as sources of side reactions^30^. This observation is supported by the increasingly worse electrochemical performance of highly crystalline 3D graphene foams (i.e., less defective) that were heat treated at even higher temperatures of 2000–2500 °C (Fig. S9).

To explore experimentally the origins of better Coulombic efficiency and high rate performance in H_2_-treated graphene, we perform electrochemical impedance spectroscopy measurements of three samples after 35 charge/discharge cycles, Nyquist plots of which are shown in Fig. 2d. The most salient feature of the H_2_-treated sample is a drastic reduction of charge transfer resistance vis-à-vis the other two samples, as manifested by a markedly smaller semi-circle at high frequencies for the GNF-1050C-H sample. Randles equivalent circuit modelling (Fig. S10 and Table S1) confirms this observation and suggests that double layer capacitance and electrolyte resistance of all three materials remain largely unchanged. This behaviour hints at possible roles of hydrogen in mediating charge-transfer and ion insertion processes during lithium binding/storage; e.g., by modifying the nature of interactions between graphitic layers. Another important observation is the drastically increased solid-electrolyte interphase (SEI) resistance in GNF-1600C, due to higher degree of graphitization at higher annealing temperatures^31^.

### Capacitive contribution

**Figure 3:**
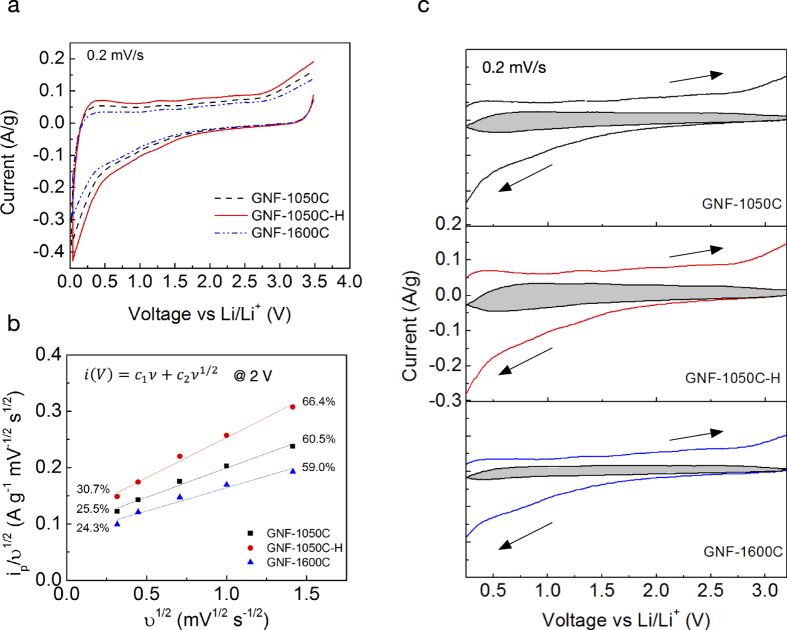
Capacitive contributions. (**a**) C-V curves of three GNF samples at a scan rate of 0.2 mV/s. (**b**) The determination of capacitive and diffusion-controlled current contributions at certain sweep rates at 2V. (**c**) The capacitive contribution (grey area) in the voltage window of 0.25–3.2V at 0.2 mV/s. The percentage capacitive contributions are 28.7%, 30.9%, and 23.0% for GNF-1050C, GNF-1050C-H, and GNF-1600C, respectively.

More information related to lithium storage kinetics of our 3D GNFs is revealed by cyclic voltammetry (CV) data shown in Fig. 3. At a constant CV scan rate of 0.2 mV/s, Fig. 3a, we observe that the H_2_-treated sample exhibits the largest current density, whereas the GNF-1600C sample has the smallest. Assuming that the current (*i*) follows a power law relation with the sweep rate (ν), we have^32^


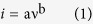


where a and b are materials-dependent variables. By using a ν range of 0.1–2 mV/s, we derive a b-value of 0.72 for the GNF-1050C, which increases to 0.75 for the GNF-1050C-H but decreases to 0.70 for the GNF-1600C, Fig. 3b. As b-values of 0.5 and 1 represent currents controlled by linear diffusion processes and surface or capacitive processes, respectively, the observation of intermediate b-values suggests both types of processes are active during lithiation. This is expected given the high surface area of the 3D GNFs used in this work. We can gain some additional physical insight by introducing a simplified model in which the two charge storage channels are functionally independent, in which case we can follow the formulation suggested in earlier studies^33^ that expresses the current (*i*) response at a given voltage (*V*) as a linear sum:


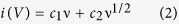


Here, c_2_ν^1/2^ describes diffusion-controlled (battery-like) contributions and c_1_ν encompasses remaining contributions that behave in a capacitive fashion. We emphasize that the “capacitive” contribution (c_1_ν) could encompass a variety of possible processes that are not diffusion-controlled, including double layer capacitance, pseudocapacitance, and general kinetically fast charge storage at accessible sites. By determining c_1_ and c_2_, the individual contributions of diffusion-controlled and capacitive mechanisms within the independent model can be estimated at each sweeping rate, as illustrated in Fig. 3b. The capacitive contribution for each material increases with sweeping rate, as one may expect. One of the most intriguing observations in Fig. 3b, however, is the enhanced capacitive contribution (and correspondingly decreased diffusion-controlled contribution) in the H_2_-treated sample compared with the other two samples. For example, at the highest sweeping rate of 2 mV/s, we find that 66.4% of lithium storage comes from capacitive mechanisms in GNF-1050C-H versus 60.5% and 59% for GNF-1050C and GNF-1600C, respectively. At a fixed scan rate of 0.2 mV/s, we observe a similar trend for three materials in the voltage window of 0.25–3.2 V, Fig. 3c. Although the two-channel independent charge storage model upon which Eqn. 2 is based neglects the full complexity of actual system, it illustrates the substantially higher capacitive contribution after H_2_ treatment, which is one of the main causes why GNF-1050C-H has the highest rate capacity.

## Discussion

### Atomistic mechanisms of high-rate capacity

The specific origin of the enhanced capacitive contribution upon H_2_ treatment is not directly discernible. However, as previously discussed, no double layer capacitive enhancement is observed in the equivalent circuit models of Fig. 2d. Furthermore, the differential capacity in the inset of Fig. 2b reveals no obvious additional redox peaks that might signal new pseudocapacitive features; instead, we observe a general enhancement and shift of existing peaks towards lower voltage onsets. The results are therefore consistent with universally faster kinetics for Li diffusion and incorporation, eliminating diffusion-related bottlenecks at high rates. Experimentally, we may conclude that atomic hydrogen helps primarily to increase diffusion kinetics and reversibility rather than the density of storage sites. In other words, the capacity increases can be attributed chiefly to the increased accessibility and improved rate performance of the electrode. This is supported by the enhanced capacitive contributions in Fig. 3, as well as the fact that the capacity improvements over the untreated sample increase with current density as expected for a kinetically driven process (Fig. 2b). Moreover, the decrease in the available sites upon first cycle in Fig. 2a excludes the possibility of increased storage sites from hydrogen treatment. Rather, the kinetic improvement is almost certainly linked to a morphological change in the electrode, since the total amount of hydrogen incorporation is relatively low (Fig. S7) and yet induces a change capable of significantly affecting the behaviour of a large number of Li^+^ ions simultaneously. The Nyquist plot in Fig. 2d offers valuable insight into this effect, since it indicates a decrease in the charge-transfer resistance and a simultaneously shortened diffusion pathway (demonstrated by the steep slope of the Warburg-like element at lower frequencies, indicative of finite diffusion). Importantly, the morphological change is accompanied by a chemical signature associated with a decrease in the aromatic ring structure of graphene. This is consistent with the XAS data in Fig. 1d that point to a reduction in *sp*^2^ carbon character, as expected from disruptions in ring structures or loss of surface carbon. At the same time, the Raman data (Fig. 1b) indicate that these chemical changes do not appreciably affect the average domain size, implying the hydrogen-induced modification occurs at sites that are already defective (e.g., non-hexagonal rings or chemically functionalized carbon structures, likely concentrated at domain boundaries). Our control experiments provide further evidence that the effect of hydrogen incorporation into graphene is related to the presence of structural or chemical defects, as we observe little performance enhancement when further annealing GNF-1600C in a H_2_ environment (SI, Fig. S11).

**Figure 4:**
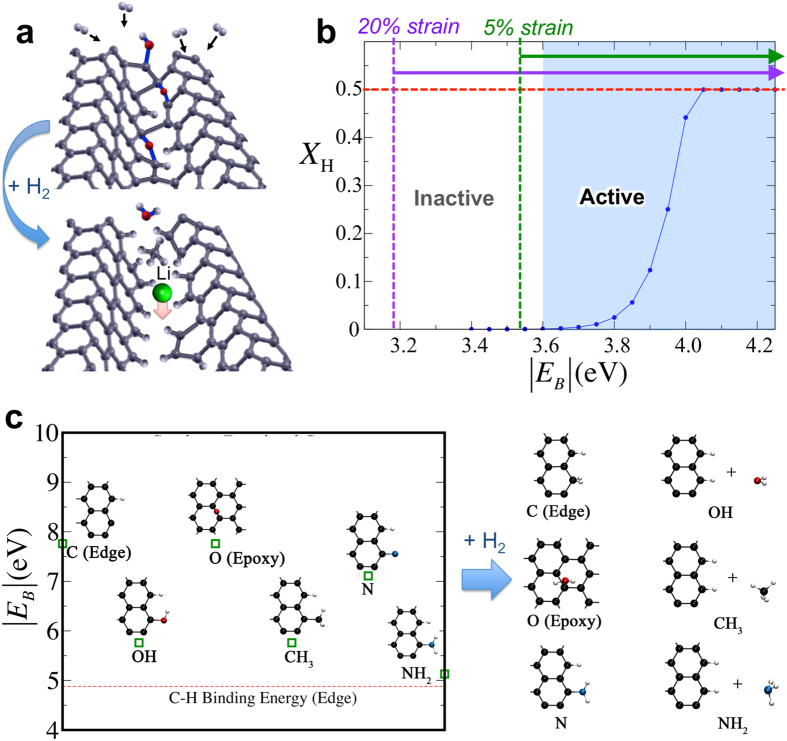
Atomistic mechanisms of dissociative hydrogenation. (**a**) Schematic of proposed mechanism for enhanced rate performance with H_2_ treatment, in which H_2_ dissociates at strained, functionalized, and/or highly defective domain boundaries, terminating edges and opening up the graphene sheet for improved Li penetration. (**b**) Equilibrium fraction of available sites terminated via a dissociative hydrogenation process of “hot” H_2_ hydrogen saturation (*X*_H_) as a function of the C-H bond formation energy at the site (*E*_B_). The ranges of *E*_B_ leading to active dissociative hydrogenation are highlighted for unstrained graphene (blue region), as well as with 5% strain (green dotted line and arrow) and 20% strain (purple dotted line and arrow) based on values in Ref. 37. The red dotted line indicates full saturation of binding sites considered in the model. (**c**) Bond formation energies *E*_B_ (per H) for the dissociative reaction of H_2_ with candidate edge functional groups with energetics sufficient for full saturation. The right side shows the corresponding hydrogenation products. The red dotted line represents hydrogenation of a reactive zigzag edge.

Based on the above important observations, we propose that hydrogen tends to “attack” high-energy structurally and chemically defective sites at domain boundaries. The reaction at the domain boundary follows three possible pathways: (1) cleavage of strained C-C bonds; (2) etching via successive reduction and hydrogenation of energetic carbon (e.g., to methane, ethane, ethane/ethylene, etc.); (3) reduction and hydrogenation of oxygen- or nitrogen-containing functional groups (e.g., to water or ammonia). In each case, the process is accompanied by H-termination of dangling carbon bonds. The broken bonds alleviate local strain and open up H-passivated nanopores and edges in the framework without creating additional traps, thus improving the accessibility of Li to regions that were previously kinetically restricted (i.e., limited to intercalation processes from the edges). This mechanism is illustrated schematically in Fig. 4a, and fits our multiple criteria of a chemically induced morphological change connected to the breakup of *sp*^2^ carbon structures that would lead to improved kinetics.

**Figure 5:**
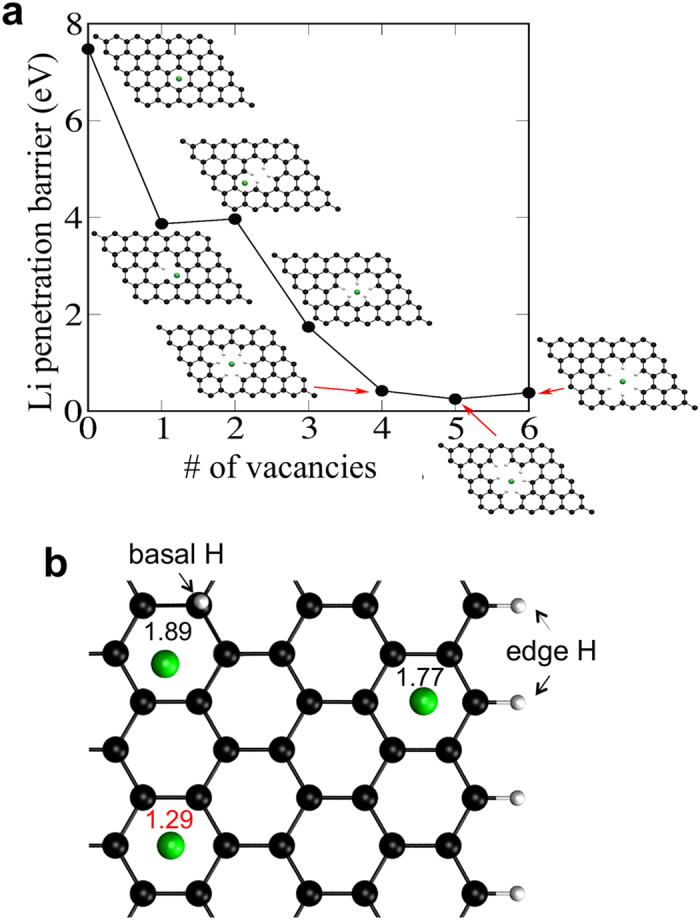
Mechanisms of high-rate capacity enhancement. (**a**) Estimated barrier for Li penetration through a graphene sheet perforated with different H-terminated pore sizes, demonstrating kinetic enhancement. (**b**) Binding energies (eV) of Li (green) at a pristine site (lower left), compared with binding near H atoms located at basal (top) and edge (right) sites where additional capacity can be activated.

Notably, the mechanism in Fig. 4a explains several observed features in the spectroscopic data. Since hydrogen preferentially “attacks” high-energy sites that are already defective, there would be no change to the domain size, consistent with the fixed D/G ratio in the Raman signature. In addition, the G-band (*E*_2g2_) Raman shift (Fig. 1b) can be explained by the increased heterogeneity at the domain boundaries, where distortions in *sp*^2^ carbon bonds would allow scattered phonons to probe non-zone centre regions of the Brillouin Zone^23^. Similarly, the slight D-band Raman shift (Fig. S2) is consistent with stabilization of medium-range order in the π manifold afforded by strain alleviation and hydrogenation at domain boundaries. The XAS spectra (Fig. 1d) can be accounted for by considering the loss of carbon due to etching, as well as a change in the local strain state of C-C bonds near the domain boundaries. The robustness of our proposed mechanism is explored through a combination of first-principles density functional theory (DFT) calculations and thermodynamic modelling (Figs 4 and 5), as discussed below and detailed in the Methods. We emphasize that the experimental results, along with our reported mechanisms, hint at the importance of *both* defective sites *and* hydrogen on the electrochemical performance of graphene.

### Mechanisms of dissociative hydrogenation

First, we demonstrate the possibility of dissociative hydrogenation (*i.e.*, dissociation of molecular H_2_ and chemisorption of atomic H) at defective sites on the graphene surface at 400 °C. According to a “hot hydrogen” model described in Ref. 34, as energetic H_2_ molecules at the tail of the thermal distribution collide with the graphene surface, the proximity of their approach alleviates the need to overcome the H_2_ high dissociation barrier (~4.12 eV)^34^ as long as an energetically favourable defective binding site is present. The induced polarization of H_2_ near defective sites may also assist in low-temperature dissociation^35^,^36^. Accordingly, the necessary energetics for dissociation H_2_ on defective binding sites need only be on the order of the bond energy difference between H in H_2_ and in defective graphene. Using relevant DFT energetics, combined with key information about our experimental conditions (Methods and SI, Fig. S12), we perform thermodynamic calculations to assess the extent of the dissociative hydrogenation process. Figure 4b shows that a 4.0 at.%-H_2_ mixture at 400^o^C will generate non-negligible H concentrations at defective binding sites, provided the binding of atomic H is sufficiently strong (see the “active” regime in the figure). Appreciable surface diffusion of atomic H can also be activated at 400 ^o^C (see Fig. S13), suggesting the additional possibility of segregation of adsorbed surface H to high-energy domain boundaries.

### Identification of candidate binding sites for dissociative hydrogenation

Importantly, the result in Fig. 4b gives an energetic lower bound for participation in dissociative hydrogenation. In particular, the defective binding sites must be exothermic for atomic H by at least Δ*E* ~ 3.60 eV. Note that in addition to energies associated with changes to chemical bonds (*E*_B_), Δ*E* may include strain energy dissipation (Δ*E*_strain_) arising from the relaxation of highly curved or strained configurations at the attack sites^37^, i.e., Δ*E* = *E*_B_ + Δ*E*_strain_. The existence of such strained regions in our graphene samples is supported by direct experimental observation of rippled/twisted structures (Fig. 1a and S1). Assuming that Δ*E*_strain_ could account for ~0.07–0.42 eV per binding site (based on the strain energy of a rippled graphene sheet induced by 5% to 20% compressive strain^37^; larger strains will further widen this window), we conclude that defective sites with *E*_B_ > 3.53 eV (assuming 5% strain) or >3.18 eV (assuming 20% strain) are possible candidates for dissociative hydrogenation (Fig. 4b).

Figure 4c illustrates some of the chemical processes that could lead to the formation of σ-type C-H bonds with values of *E*_B_ fitting Fig. 4b. The computed energy of H binding on a free nanoribbon edge is 4.88 eV (Fig. 4c), well within our window (by contrast, the value for basal H binding on graphene is only 0.81 eV, prohibiting dissociative hydrogenation at conventional surface sites). The target range for *E*_B_ is also compatible with H binding at the edges of multi-vacancy clusters (e.g., the 5-vacancy cluster shown in Fig. 5a has an edge H binding energy of 3.55 eV). Several other possible reactions involving successive hydrogenation of domain-edge carbon atoms and of residual oxygen or nitrogen are shown in Fig. 4c; such defect motifs may be introduced during processing or upon reaction with environmental agents^38^. Each is highly exothermic and fits the criterion of Fig. 4b, with reactive sites passivated by the addition of dihydrogen. Scenarios include H_2_ dissociation to etch away atomically rough edges by formation of CH_4_, or else the replacement of O-, OH-, or NH_x_-containing groups with H_2_O or NH_3_, hydrogenating any remaining dangling bonds in the process. Note that molecular products of these reactions (e.g., H_2_O, CH_4_, and NH_3_) would be released at low pressure or elevated temperatures, effectively conserving the total amount of hydrogen in the system; this may explain the largely similar H concentrations before and after hydrogen treatment detected in high vacuum conditions (<~10^−6^ Torr) by ERDA and implied in the XAS results. To conclude, we find that functionalized edges, multi-vacancy complexes, and strained non-hexagonal rings — likely to be concentrated at strained domain boundaries — all constitute probable sites for dissociative hydrogenation.

### Enhanced Li penetration at hydrogenated boundaries

To test the degree of kinetic enhancement associated with hydrogenation of multi-vacancy complexes and domain boundaries according to the mechanism in Fig. 4a, we estimated the DFT barrier associated with Li diffusion through hydrogen-terminated perforations of different sizes within a pristine graphene sheet (Methods). As shown in Fig. 5a, we find that very low barriers (~0.3 eV) are obtained even for relatively small perforations (>3 atom vacancies) provided the bonds are hydrogen passivated. Accordingly, even slight opening of the lattice due to strain alleviation or carbon etching at strained domain boundaries could have significant impacts on the diffusion kinetics, with passivation mitigating any chemical or electrostatic interaction with Li^+^ that could otherwise lead to irreversible trapping. We point out that increases in the interlayer spacing upon insertion and binding of surface H between graphene layers could further aid intercalation near the perforations, as explored theoretically in SI, Fig. S14; however, analysis of X-ray diffraction patterns provides little evidence of systematic increases in the layer spacing upon hydrogenation of our samples (Fig. S5).

### Additional reversible capacity

Although the dominant effect of hydrogenation is improved kinetics associated with enhanced surface capacitive behaviour (suggested by the results in Fig. 3 and the mechanism of Fig. 4a), our calculations also suggest that the addition of hydrogen at a dangling-bond or basal-plane site will have a secondary positive impact on overall capacity by activating nearby reversible binding sites (Fig. 5b and SI, Supplementary discussion, Figs S15–S19). On pristine graphene, it is well established that Li^+^ binding is too weak for practical use as an anode. Interestingly, the addition of hydrogen enhances the adsorption of Li^+^ in the hexagonal rings nearest the hydrogen (specifically, the near-edge ring for a dangling-bond edge site or a nearest-neighbour ring on the opposing side of a basal-plane site, as shown in Fig. 5b). The resulting Li binding energies are within a target range that encompasses adequate stability against Li agglomeration (*E*_B_ > 1.6 eV) while maintaining reversibility under practical cycling conditions. The introduction of curvature would further stabilize such sites. We emphasize that these mechanisms can only add reversible capacity from local sites nearest the hydrogen, and would therefore have a negligible effect on overall kinetics. Accordingly, they cannot represent the dominant mechanism for our reported high-rate capacity increase, but may nonetheless provide a measureable contribution.

In summary, we have investigated the “hydrogen effect” in the electrochemical performance of defective and highly crystalline 3D GNFs, through a series of heat treatment experiments and computer simulations. Defects (e.g., vacancies, edges) and hydrogen are found to play an integral role in helping enhance the lithiation kinetics, leading to high rate capacity in hydrogen treated graphene materials. This behaviour appears universal in all hydrogen treated samples. Among several beneficial roles of hydrogen, the binding of hydrogen along domain boundaries is considered as an effective pathway to help achieve high-rate performance 3D GNF electrodes, as it can substantially lower the barrier for Li^+^ penetration. Our studies further reveal a synergy between hydrogen and defects, in that hydrogenation becomes ineffective in highly crystalline GNFs due to a substantial loss of defective sites for hydrogen binding. This connection underscores the desirability of synthetic avenues or processing treatments that provide some residual native defects or O- and N-containing functional groups as target sites for hydrogenation in graphene derivatives. Questions remain as to how to optimize defect density and hydrogen incorporation in graphene materials in order to achieve high energy density and high power density for LIB applications.

## Methods

### Fabrication of graphene nanofoams (GNFs) and heat treatment

GNFs were synthesized by gelation of graphene oxide followed by supercritical drying and high temperature pyrolysis. 400 mg single-layer graphene oxide powder (~300–800 nm in lateral dimensions, Cheap Tubes inc.) were suspended in 20 mL deionized (DI) water (resistivity of 18.2 MΩ). After adding 4.22 ml concentrated ammonium, the suspension was sonicated overnight at 15 °C. The obtained slurry was poured into a rubber mould (diameter of 12.5 cm and thickness of 1 mm) and sealed between glass slides. The slurry-filled mould was immersed in DI water and placed in an oven for gelation at 80 °C. The wet gel was washed in DI water, followed by acetone. The acetone was exchanged with liquid CO_2_ in a critical point drier overnight. The drier was heated to 50 °C and the pressure was adjusted to maintain ~1500 psi to exceed the critical point of CO_2_. The gel was held at this state for 2 h, after which the drier was slowly vented to ambient pressure to remove dried gels. The obtained dried gels were placed in a tube furnace for carbonization at 1050 °C for 4 h in N_2_. The GNF sample was further annealed at 400 °C in 4 at% H_2_/Argon for 24 h to obtain GNF-1050C-H, or pure H_2_ for 4 h to obtain GNF-1050C-H2. To increase crystallinity, GNF-1050C was further annealed at 1600 °C in argon, 2000 °C in helium, and 2500 °C in helium for 4 h.

### SEM and TEM characterizations

Scanning electron microscopy (SEM) was conducted using a JOEL JSM-7401F field-emission SEM under secondary electron imaging (SEI) mode with an accelerating voltage of 20 kV and a beam current of 20 mA. The cross sectional GNFs with fresh fracture surface was examined. Transmission electron microscopy (TEM) was performed on a Philips CM30 field-emission TEM. Samples were prepared by manually crashing GNF samples onto a Lacey carbon supported copper grids (200 mesh, Ted Pella, Inc.).

### Porosimetry

The porosimetry was determined by Brunauer-Emmett-Teller (BET) and Barrett-Joyner-Halenda (BJH) methods in nitrogen adsorption/desorption experiments using an ASAP 2020 Surface Area Analyser (Micromeritics Instrument Corporation). GNF samples with mass of 10 mg to 20 mg were preheated at 300 °C under N_2_ flow for 24 h to remove moisture.

### Raman spectroscopy

Raman spectra were collected using three laser excitation wavelengths, 473 nm, 532 nm, and 633 nm. The spot size was 1.0–2.5 μm, and the laser beam power was 1–2 mW. Table S2 lists the detailed spot size, power, and spectral resolution of each excitation laser wavelength for the measurements. At least three spectra were collected for each sample. To analyse the position and intensity of D, G, and D’ bands, the background or raw spectra data was subtracted by a linear function and the peaks were deconvolved by multi-peak fitting using Lorentzian functions. The domain size or crystallite size (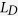) was estimated from I(D)/I(G) ratio using the following equation:


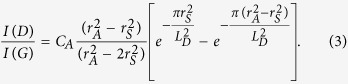


Here 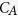 is the weighing parameter of an active area (A) that can be expressed as 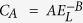, where A = (160 ± 48) eV, B = 4, and 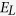 = 1.96 eV. 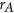 and 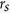 are two length scale parameters that determine the region of D band scattering, which were fitted to be 3 nm and 1 nm, respectively.

### X-ray absorption spectroscopy (XAS)

XAS measurements were performed at beamline (BL) 8.0.1.1 of the Advanced Light Source, Lawrence Berkeley National Laboratory. Carbon K-edge XAS data was recorded in the total electron yield (TEY) mode over a spectral range of 280–330 eV, with the sample angled at 45°with respect to the incident x-ray beam. Energy calibration of BL8.0.1.1 was achieved via reference to the C(1s) → π* resonance for a freshly cleaved sample of highly oriented pyrolytic graphite (285.38 eV)^39^. All XAS data was normalized to both the incident x-ray flux, I_0_, and the absorption edge step. I_0_ was measured concurrently with the XAS signal via the drainage current from a gold grid located upstream of the experimental sample and the absorption edge step was taken as the difference in absorbance in the pre-edge (280 eV) and post-edge (330 eV) regions of the XAS spectrum.

### X-ray diffraction (XRD)

XRD was carried out using Bruker AXS D8 ADVANCE X-ray diffractometer with Cu radiation source operated at 40 kV and 40 mA. 2θ scan was conducted from 10° to 90° with 0.02° steps and 2 s counting time per step.

### Elastic recoil detection analysis (ERDA) and Rutherford back scattering (RBS)

Sample was mounted on a Si piece. Mounting and sample transfer were completed in N_2_ environment. The H-content in GNF was determined by elastic recoil detection analysis (ERDA)^40^ with 3 MeV ^4^He^+^ ions. During the ERDA experiment, the sample normal direction was tilted by 70° with respect to the incident He beam. H atoms recoiled into a surface barrier detector at 150° were measured. The detector is covered with a 13-um thick carbon foil that was used to filter out the forward-scattered He ions. The oxygen content was measured by Rutherford backscattering spectrometry (RBS) with a 2 MeV ^4^He^+^ beam incident normal to the sample surface. The detector located at 164° from the incident beam direction was used to register backscattered He ions. Both ERDA and RBS spectra were analysed with the RUMP code^41^ with a cross-section for the ^1^H(^4^He, ^1^H) ^4^He reaction from Ref. 42 The average H-content is calculated by assuming linear variation in the first 10 μm surface layer and constant behaviour after 10 μm.

### Electrochemical measurements

Swagelok-type Half cells were assembled in an Argon-filled glove box with GNFs as the working electrodes, lithium as the counter electrodes, two layers of Celgard 3501 porous polypropylene films as the separators, and 1 M LiPF6 in 1:1:1 (in volume) of ethyl carbonate (EC)/diethyl carbonate (DEC)/dimethyl carbonate as electrolyte. Galvanostatic charge/discharge experiments were performed on a Maccor 4304 Battery cycler (voltage window 0.01–3.5 V)^43^,^44^. Electrochemical impedance spectra (EIS) were recorded after rate jump experiments at 3.5 V using a Bio-Logic electrochemical workstation with frequencies ranging from 100 kHz to 10 mHz, and an amplitude of 5 mV. Cyclic voltammetry (CV) experiments were conducted in the voltage range from 3.5 V to 0.01 V with voltage sweep rate from 0.1–2 mV/s. All the samples for CV experiments were after 35 cycles of rate-jump tests.

### Thermodynamics calculations

We consider a graphene surface consisting of only one type of defective binding site where the energy change upon H binding is given by Δ*E*. Within the model discussed in Ref. 34, the dissociation reaction can be written as


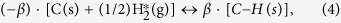


where β is the extent of reaction and 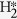 (g) represents a ‘*hot*’ hydrogen gas molecule. The moles of 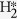 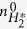 at temperature *T* is calculated by 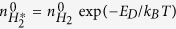, where 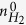 is the total moles of H_2_ molecules flowing into the chamber and *E*_*D*_ = |Δ*E*|–|*E*_B_|. The total energy variation upon dissociative hydrogenation is computed as follows:


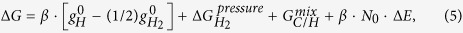


where 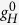 and 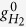 are the molar Gibbs free energies of H atoms and H_2_ molecules, respectively, at the standard state, and *N*_0_ is Avogadro’s number. 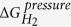 is the free energy change upon reaction due to the partial pressure variation (accounting for zero-point, entropy, and enthalpy contributions^45^) and 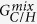 is the free energy of mixing for the hydrogenated graphene. Ideal-gas and ideal-mixing behaviour are assumed. Using Eq. (5) and the phase fraction definitions in Table S3, we determine the β that minimizes ΔG to obtain the thermodynamically stable H content. See Supplementary Methods for further details.

### First-principles simulations

We use density functional theory implemented in VASP^46^ with the projector augmented wave method^47^ and the Perdew-Burke-Erzenhof (PBE) exchange-correlation functional^48^. Basal-plane Li adsorption was simulated using a 6 × 6 graphene supercell. Edge-site Li adsorption was simulated using a zigzag graphene nanoribbon in a 72-atom orthorhombic unit cell, edge-terminated with 12 hydrogen atoms. A 400 eV plane-wave cutoff was used. Brillouin zone sampling for the basal (nanoribbon) case was based on a 5 × 5 × 1 (5 × 1 × 1) Monkhorst-Pack *k*-point mesh. Periodic boundary conditions were used, with a 20 Å vacuum layer inserted perpendicular to the planes and a dipole correction applied. Gaussian smearing was used with a smearing width of 0.03 eV. Forces were converged to <1 × 10^−2^ eV/Å. For calculation of surface diffusion barriers (Fig. S11), the nudged elastic band (NEB) method^49^ was used. Li binding energies were calculated as 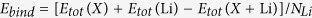, where Li, *X*, and *X* + Li refer to an isolated Li atom and to Li-free and Li-adsorbed substrates, respectively, and *N*_Li_ is the number of Li atoms in the cell. Vacancy complexes were generated by terminating dangling bonds with hydrogen within the graphene plane. The energy barrier for Li penetration through the vacancy complexes was estimated as the energy differences between constrained optimizations of two Li positions: (1) above the largest opening in the graphene sheet at a distance of 1.72 Å (the equilibrium binding distance of Li on the basal plane of pristine graphene); and (2) in the plane of graphene at the centre of the largest opening.

## Additional Information

**How to cite this article**: Ye, J. *et al.* Universal roles of hydrogen in electrochemical performance of graphene: high rate capacity and atomistic origins. *Sci. Rep.* **5**, 16190; doi: 10.1038/srep16190 (2015).

## Supplementary Material

Supplementary Information

## Figures and Tables

**Table 1:** Processing conditions of graphene nanofoams (GNFs) samples used in this study.

Sample ID	Processing conditions
GNF-1050C	Pyrolyzed at 1050 °C in N_2_ (99.99^+^%), 4hrs, reference sample
GNF-1050C-H	GNF-1050C sample further heat treated at 400 °C, 100-sccm flow of 4at% H_2_/Ar, 24 hrs
GNF-1050C-H2	GNF-1050C sample further heat treated at 400 °C, 100-sccm flow of 100% H_2_, 4 hrs
GNF-1600C	GNF-1050C sample further heat treated at 1600 °C, Ar atmosphere, 4 hrs
GNF-1600C-H	GNF-1600C sample further heat treated at 400 °C, 100-sccm flow of 4at% H_2_/Ar, 24 hrs
GNF-2000C	GNF-1050C sample further heat treated at 2000 °C, He atmosphere, 4 hrs
GNF-2500C	GNF-1050C sample further heat treated at 2500 °C, He atmosphere, 4 hrs

**Table 2:** Surface area and pore volume of three key GNF samples for electrochemical performance measurements.

Samples	Specific surface area (m^2^/g)	Peak pore diameter (nm)	Pore volume (cm^3^/g)
GNF-1050C	1340	6.0	4.0
GNF-1050C-H	1329	4.3	4.5
GNF-1600C	1067	5.2	3.9

## References

[b1] Raccichini R., Varzi A., Passerini S. & Scrosati B. The role of graphene for electrochemical energy storage. Nat. Mater. 14, 271–279, 10.1038/NMAT4170 (2015). 25532074

[b2] Bonaccorso F. *et al.* Graphene, related two dimensional crystals, and hybrid systems for energy conversion and storage. Science 347, 1246501, 10.1126/science.1246501 (2015). 25554791

[b3] Xu Y. *et al.* Holey graphene frameworks for highly efficient capacitive energy storage. Nat. Commun. 5, 4554, 10.1038/ncomms5554 (2014). 25105994

[b4] Yang X., Cheng C., Wang Y., Qiu L. & Li D. Liquid-Mediated Dense Integration of Graphene Materials for Compact Capacitive Energy Storage. Science 341, 534–537, 10.1126/science.1239089 (2013). 23908233

[b5] Zhu Y. *et al.* Carbon-Based Supercapacitors Produced by Activation of Graphene. Science 332, 1537–1541, 10.1126/science.1200770 (2011). 21566159

[b6] Li N., Chen Z., Ren W., Li F. & Cheng H.-M. Flexible graphene-based lithium ion batteries with ultrafast charge and discharge rates. Proc. Natl. Acad. Sci. USA 109, 17360–17365, 10.1073/pnas.1210072109 (2012). 23045691 PMC3491507

[b7] Xu Y. *et al.* Solvated graphene frameworks as high-performance anodes for lithium-ion batteries. Angew. Chem. 127, 1–7 (2015). 10.1002/anie.201500677 25756737

[b8] Park S. & Ruoff R. S. Chemical methods for the production of graphenes. Nat. Nanotech. 4, 217–224, 10.1038/nnano.2009.58 (2009). 19350030

[b9] Hernandez Y. *et al.* High-yield production of graphene by liquid-phase exfoliation of graphite. Nat. Nanotech. 3, 563–568, 10.1038/nnano.2008.215 (2008). 18772919

[b10] Li Z. *et al.* Effect of airborne contaminants on the wettability of supported graphene and graphite. Nat. Mater. 12, 925–931, 10.1038/nmat3709 (2013). 23872731

[b11] Elias D. C. *et al.* Control of Graphene’s Properties by Reversible Hydrogenation: Evidence for Graphane. Science 323, 610–613, 10.1126/science.1167130 (2009). 19179524

[b12] Ryu S. *et al.* Reversible Basal Plane Hydrogenation of Graphene. Nano Lett. 8, 4597–4602, 10.1021/nl802940s (2008). 19053793

[b13] Balog R. *et al.* Bandgap opening in graphene induced by patterned hydrogen adsorption. Nat. Mater. 9, 315–319, 10.1038/nmat2710 (2010). 20228819

[b14] Dahn J. R., Zheng T., Liu Y. H. & Xue J. S. Mechanisms for llithium insertion in carbonaceous materials. Science 270, 590–593, 10.1126/science.270.5236.590 (1995).

[b15] Cabria I., Lopez M. J. & Alonso J. A. Enhancement of hydrogen physisorption on graphene and carbon nanotubes by Li doping. J. Chem. Phys. 123, 10.1063/1.2125727 (2005). 16351307

[b16] Dimitrakakis G. K., Tylianakis E. & Froudakis G. E. Pillared Graphene: A New 3-D Network Nanostructure for Enhanced Hydrogen Storage. Nano Lett. 8, 3166–3170, 10.1021/nl801417w (2008). 18800853

[b17] Vargas O. A., Caballero A. & Morales J. Can the performance of graphene nanosheets for lithium storage in Li-ion batteries be predicted? Nanoscale 4, 2083–2092, 10.1039/c2nr11936f (2012). 22358220

[b18] Ye J. C. *et al.* Enhanced electrochemical performance of ion-beam-treated 3D graphene aerogels for lithium ion batteries. Carbon 85, 269–278, 10.1016/j.carbon.2014.12.097 (2015).

[b19] Lee E. & Persson K. A. Li Absorption and Intercalation in Single Layer Graphene and Few Layer Graphene by First Principles. Nano Lett. 12, 4624–4628, 10.1021/nl3019164 (2012). 22920219

[b20] Liu Y., Artyukhov V. I., Liu M., Harutyunyan A. R. & Yakobson B. I. Feasibility of Lithium Storage on Graphene and Its Derivatives. J. Phys. Chem. Lett. 4, 1737–1742, 10.1021/jz400491b (2013). 26282987

[b21] Liu Y., Wang Y. M., Yakobson B. I. & Wood B. C. Assessing Carbon-Based Anodes for Lithium-Ion Batteries: A Universal Description of Charge-Transfer Binding. Phys. Rev. Lett. 113, 028304, 10.1103/PhysRevLett.113.028304 (2014). 25062244

[b22] Worsley M. A. *et al.* Synthesis and characterization of highly crystalline graphene aerogels. Acs Nano 8, 11013–11022, 0.1021/nn505335u (2014). 25283720 10.1021/nn505335u

[b23] Ferrari A. C. *et al.* Raman spectrum of graphene and graphene layers. Phys. Rev. Lett. 97, 187401, 10.1103/PhysRevLett.97.187401 (2006). 17155573

[b24] Narula R. & Reich S. Double resonant Raman spectra in graphene and graphite: A two-dimensional explanation of the Raman amplitude. Phys. Rev. B 78, 165422, 10.1103/PhysRevB.78.165422 (2008).

[b25] Matthews M. J., Pimenta M. A., Dresselhaus G., Dresselhaus M. S. & Endo M. Origin of dispersive effects of the Raman D band in carbon materials. Phys. Rev. B 59, R6585–R6588, 10.1103/PhysRevB.59.R6585 (1999).

[b26] Eckmann A. *et al.* Probing the Nature of Defects in Graphene by Raman Spectroscopy. Nano Lett. 12, 3925–3930, 10.1021/nl300901a (2012). 22764888

[b27] Zandiatashbar A. *et al.* Effect of defects on the intrinsic strength and stiffness of graphene. Nat. Commun. 5, 3186, 10.1038/ncomms4186 (2014). 24458268

[b28] Ma Y. *et al.* Core excitons and vibronic coupling in diamond and graphite. Phys. Rev. Lett. 71, 3725–3728, 10.1103/PhysRevLett.71.3725 (1993). 10055056

[b29] Coffman F. L. *et al.* Near-edge x-ray absorption of carbon materials for determining bond hybridization In mixed sp2/sp3 bonded materials. Appl. Phys. Lett. 69, 568–570, 10.1063/1.117789 (1996).

[b30] Wu Z.-S., Ren W., Xu L., Li F. & Cheng H.-M. Doped Graphene Sheets As Anode Materials with Superhigh Rate and Large Capacity for Lithium Ion Batteries. Acs Nano 5, 5463–5471, 10.1021/nn2006249 (2011). 21696205

[b31] Goodenough J. B. & Kim Y. Challenges for Rechargeable Li Batteries. Chem. Mater. 22, 587–603, 10.1021/cm901452z (2010).

[b32] Augustyn V. *et al.* High-rate electrochemical energy storage through Li^+^ intercalation pseudocapacitance. Nat. Mater. 12, 518–522, 10.1038/nmat3601 (2013). 23584143

[b33] Augustyn V., Simon P. & Dunn B. Pseudocapacitive oxide materials for high-rate electrochemical energy storage. Energy Environ. Sci. 7, 1597–1614, 10.1039/c3ee44164d (2014).

[b34] Mikoushkin V. M. *et al.* Graphene hydrogenation by molecular hydrogen in the process of graphene oxide thermal reduction. Appl. Phys. Lett. 102, 4, 10.1063/1.4793484 (2013).

[b35] Kim B. H. *et al.* N-type graphene induced by dissociative H-2 adsorption at room temperature. Sci. Rep. 2, 10.1038/srep00690 (2012). PMC3457033 23012645

[b36] Ulman K., Bhaumik D., Wood B. C. & Narasimhan S. Physical origins of weak H-2 binding on carbon nanostructures: Insight from ab initio studies of chemically functionalized graphene nanoribbons. J. Chem. Phys. 140, 174708, 10.1063/1.4873435 (2014). 24811656

[b37] Dutta D., Wood B. C., Bhide S. Y., Ayappa K. G. & Narasimhan S. Enhanced Gas Adsorption on Graphitic Substrates via Defects and Local Curvature: A Density Functional Theory Study. J. Phys. Chem. C 118, 7741–7750, 10.1021/jp411338a (2014).

[b38] Acik M. *et al.* The Role of Intercalated Water in Multilayered Graphene Oxide. Acs Nano 4, 5861–5868, 10.1021/nn101844t (2010). 20886867

[b39] Batson P. E. Carbon-1s near-edge-adsorption find-structure in graphite. Phys. Rev. B 48, 2608–2610, 10.1103/PhysRevB.48.2608 (1993). 10008656

[b40] Tirira J., Serruys Y. & Trocellier P. Forward Recoil Spectrometry. (Springer: US,, 1996).

[b41] Doolittle L. R. Nuclear Instruments and Methods in Physics Research Section B 9, 344 (1985). 10.1016/0168-583x(85)90137-5 11539755

[b42] Baglin J. E. E., Kellock A. J., Crockett M. A. & Shih A. H. Nuclear Instruments and Methods in Physics Research Section B 64, 469 (1992).

[b43] Bhardwaj T., Antic A., Pavan B., Barone V. & Fahlman B. D. Enhanced Electrochemical Lithium Storage by Graphene Nanoribbons. J. Am. Chem. Soc. 132, 12556–12558, 10.1021/Ja106162f (2010). 20731378

[b44] Yoo E. *et al.* Large Reversible Li Storage of Graphene Nanosheet Families for Use in Rechargeable Lithium Ion Batteries. Nano Lett. 8, 2277–2282, 10.1021/Nl800957b (2008). 18651781

[b45] Chase M. W. Jr. NIST-JANAF Themochemical Tables. J. Phys. Chem. Ref. Data Monograph 9, 1–1951 (1998).

[b46] Kresse G. & Furthmuller J. Efficient iterative schemes for ab initio total-energy calculations using a plane-wave basis set. Phys. Rev. B 54, 11169–11186, 10.1103/PhysRevB.54.11169 (1996). 9984901

[b47] Blochl P. E. Projector augmented-wave method. Phys. Rev. B 50, 17953–17979, 10.1103/PhysRevB.50.17953 (1994). 9976227

[b48] Perdew J. P., Burke K. & Ernzerhof M. Generalized gradient approximation made simple. Phys. Rev. Lett. 77, 3865–3868, 10.1103/PhysRevLett.77.3865 (1996). 10062328

[b49] Henkelman G., Uberuaga B. P. & Jonsson H. A climbing image nudged elastic band method for finding saddle points and minimum energy paths. J. Chem. Phys. 113, 9901–9904, 10.1063/1.1329672 (2000).

