# Supplementary material for: Universal roles of hydrogen in electrochemical performance of graphene: high rate capacity and atomistic origins
Source: Sci Rep. 2015 Nov 5;5:16190. doi: 10.1038/srep16190 (PMC4633639; doi:10.1038/srep16190)

**Supplementary Information (SI) for**

# Universal roles of hydrogen in electrochemical performance of graphene: high rate capacity and atomistic origins

Jianchao Ye[§], Mitchell T. Ong[§], Tae Wook Heo, Patrick G. Campbell, Marcus A. Worsley, Yuanyue Liu, Swanee J. Shin, Supakit Charnvanichborikarn, Manyalibo J. Matthews, Michael Bagge-Hansen, Jonathan R.I. Lee, Brandon C. Wood[*], Y. Morris Wang[*]

Physical and Life Sciences Directorate, Lawrence Livermore National Laboratory, Livermore, California 94550, USA

[§]These authors contribute equally to this work.

*Email: brandonwood@llnl.gov or ymwang@llnl.gov

**This Supplementary Information file includes:**

**Supplementary methods** (further details of thermodynamic calculations).

**Supplementary discussion** (effects of various additional hydrogenation mechanisms on Li capacity predicted from first principles).

**Supplementary Tables and Figures**: Tables S1-S3 and Figures S1-S19.

## Supplementary methods

**Further details of thermodynamics calculations.** As discussed in the Methods section of the main text, we consider the following dissociation reaction:

$$(-\beta)\cdot[\mathrm{C(s)}+(1/2)\mathrm{H}_2^*(\mathrm{g})] \leftrightarrow \beta\cdot[\mathrm{C\text{-}H(s)}], \qquad \text{(S1)}$$

where β is the extent of reaction. $\mathrm{H}_2^*(\mathrm{g})$ represents a '*hot*' hydrogen gas molecule at the tail of the Maxwell-Boltzmann distribution[34]. The amount of $\mathrm{H}_2^*$ $n_{H_2^*}^0$ at a given temperature *T* is calculated by $n_{H_2^*}^0 = n_{H_2}^0 \exp(-E_D / k_B T)$, where $n_{H_2}^0$ is the total amount of $H_2$ molecules flowing into the chamber and $E_D$=|Δ*E*|-|$E_B(H_2)$|. By analysing the reaction at β starting from $n_C^0$ and $n_{H_2^*}^0$ moles of carbon atoms and $\mathrm{H}_2^*$ molecules, respectively, we obtain the amount of each species from Eq. (S1) as shown in Table S3. We note that β has the constraints $0 \le \beta \le n_C^0$ and $0 \le \beta/2 \le n_{H_2^*}^0$, since the total amounts of participating carbon atoms and $\mathrm{H}_2^*$ molecules are limited.

In the expression for the total energy variation upon dissociative hydrogenation (Eq. 5 in the main text), the expressions for $\Delta G_{H_2}^{pressure}$ and $G_{C/H}^{mix}$, which represent the energy changes due to the partial pressure variation upon reaction and the mixing energy of the C/H mixture, respectively, are derived as:

$$\Delta G_{H_2}^{pressure} = n_{H_2^*}\cdot RT\cdot \ln P_{H_2^*,\,2} - n_{H_2^*}^0\cdot RT\cdot \ln P_{H_2^*,\,1}, \qquad \text{(S2)}$$

$$G_{C/H}^{mix} = R\cdot T\cdot[n_C^0 \ln(n_C^0/(n_C^0-\beta)) + n_H \ln((n_C^0-\beta)/\beta)], \qquad \text{(S3)}$$

where $n_{H_2^*} = n_{H_2^*}^0 - \beta/2$ and $P_{H_2^*,1}$ and $P_{H_2^*,2}$ are the partial pressures of $\mathrm{H}_2^*$ before and after the reaction, respectively. Note that we assume ideal mixing of C and H atoms at the graphene surface with a fixed number of binding C sites for $G_{C/H}^{mix}$ arising from configurational entropy and ideal behaviour of $\mathrm{H}_2^*$ gas molecules. For the initial amount of carbon atoms and hydrogen molecules in the reaction, we use $n_C^0 = 0.6409\times10^{-4}\,moles$ (for 3D graphene aerogel 250 μm thick and 7.5 mm in diameter) and $n_{H_2}^0 = 0.236\,moles$ (for 4 at.%$H_2$/Ar gas mixture, 100 *sccm*, 1 *atm*, 24 *hours*) obtained from our experimental conditions. The Gibbs free energies of the H atom ($g_H^0/N_0$) and $H_2$ molecule ($g_{H_2}^0/N_0$) at the standard state are computed as -1.116 eV and -6.760 eV, which also includes energies computed from first-principles simulations, as well as the zero-point, entropy, and enthalpy contributions of $H_2$ gas molecules at our given conditions, as taken from NIST tables. We routinely repeated the minimization process by changing Δ*E* in Eq. 5 in the main text at *T* = 400 °C to obtain Fig. 4c.

## Supplementary discussion

**Surface diffusion of H on graphene.** In Fig. S13, we explore the possibility of surface diffusion of basal H on pristine graphene at the experimental temperatures. Using NEB, the diffusion barrier between neighbouring carbon sites was found to be $E_d$ = 1.06 eV. We estimated the diffusion length using the following formula:

$$l = \sqrt{D_p \exp\left(-\frac{E_d}{k_B T}\right) \times t}, \qquad \text{(S4)}$$

where $t$ = 24 hours. $D_p$ is computed according to $D_p = \frac{1}{4}(6\nu_0) \times a_0^2$, where the prefactor ¼ represents two-dimensional diffusion, the factor of 6 accounts for the possible jump directions in the hexagonal lattice, and $a_0$ is the nearest-neighbour carbon spacing in the graphene lattice. The attempt frequency $\nu_0$ was assumed to be $10^{13}$/s. The estimated diffusion length for 24 hours of exposure is plotted as a function of temperature in Fig. S13. It is clear that at the experimental H treatment conditions of 400°C (673K) for 24 hours, surface diffusion over distances >100 nm is expected, making spontaneous segregation of H to high-energy sites at domain boundaries very likely.

**Additional possible H binding sites.** We have examined a number of possible routes for hydrogen-induced enhancement of high-rate capacity, several of which are discussed briefly in the main text. In particular, we have looked at three different mechanisms in which the presence of hydrogen to graphene could possibly enhance Li binding to the surface to create viable binding sites, as well as an intercalation mechanism in which hydrogen increases the interlayer spacing for improved Li intercalation kinetics. Details of these calculations are given below.

*I. Intercalation Mechanism for Improved Kinetics*

We have examined the possibility that hydrogen might improve the kinetics of Li intercalation into the graphitic anode material (Fig. S14). We first examined the effects of having hydrogen intercalated in between bilayer graphene. We also explored the effect of having a monovacancy and H-terminated monovacancy present in one of the graphene layers (this effect was found to be negligible). We assume that the preferred stacking of bilayer graphene is *AB* stacking, which was confirmed by calculating the interaction energy between two layers for *AA* and *AB* stacking. Upon inserting 9 hydrogen atoms uniformly on one layer of graphene between the two sheets, we found that there is a 12% increase in the interlayer spacing from 3.24 Å to 3.67 Å. In addition, this is coupled with a decrease in the overall interaction energy. Therefore, having a hydrogen-rich environment could result in intercalation of H atoms between the graphene sheets resulting in a larger interlayer spacing in which Li atoms can more easily pass through into the anode material.

*II. Hydrogen binding on basal plane*

One possible mechanism (Fig. S15) involves hydrogen binding to a basal site on the surface of graphene. As shown in Fig. 5b of the main text, Li binding to pristine graphene results in a binding energy of 1.29 eV. Binding Li to graphene with one hydrogen atom already present on the surface results in a binding energy of 1.89 eV when adding to the opposite side of the hydrogen atom (see Fig. 5b in main text), and 1.54 eV when adding to the same side as the hydrogen atom. We note that binding of Li directly on top of the H atom results in LiH formation and unbinding from the surface. Interestingly, we find that the larger binding energy is due to the charge donated by the hydrogen and not the $sp^3$ hybridization that arises from hydrogen binding to graphene. Therefore, we conclude that the presence of basal hydrogen on the surface leads to a greater preference for Li binding. Furthermore, the binding energy is not strong enough to be irreversible, so Li can unbind from the surface during cycling.

*III. Hydrogen binding on edge sites*

In another possible mechanism (Fig. S16), we have examined binding of the Li atom to an edge site that could arise in defective graphene samples. We find that if the edge site is terminated with hydrogen, the binding energy for Li to bind to the honeycomb lattice closest to the edge site is 1.77 eV (this is the value reported in Fig. 5b in the main text). Comparing this to the value of Li on pristine graphene, there is much stronger binding when the Li atom is located near an H-terminated edge site. Furthermore, adding a second Li atom at another edge site on the same side as the first Li results in an even larger binding energy of 1.86 eV. From this, we conclude the presence of an edge site with H-terminated edges results in increased preference for Li binding, which could possibly lead to greater Li storage capacity in graphitic anode materials.

*IV. Bridging hydrogen atoms*

Another possible mechanism (Fig. S17) that we explored was having bridging hydrogen atoms, which could stabilize binding of Li on the surface. We look at different positions of binding a second hydrogen atom, namely the ortho, meta and para positions with respect to the first hydrogen atom. The Li atom was placed between these two hydrogen atoms forming a bridge above the surface. The Li binding energies we calculated were 0.28, 1.51 and 1.21 eV, respectively. We see that the only case that enhances the Li binding energy is having the hydrogen atoms in the meta configuration while the Li atom binds nearby, but not between the two hydrogen atoms. This produces only a similar value to binding Li with only one hydrogen atom present. Therefore, we find that there is little improvement of Li binding by having small clusters of multiple hydrogen atoms on graphene's surface.

*V. Electronic Band Structures*

We show in Figs. S18 and S19 the band structures the effect of individual atom adsorption of H and Li on graphene. As shown in Fig. S18, the effect of the H atom on graphene introduces a low lying band near the Fermi level due to the H atom being chemisorbed on the surface of

graphene resulting in $sp^3$ hybridization. In contrast, we see the effect of Li on graphene is shifting the Fermi level higher in energy as a result of n-type doping of graphene. The combination of these two effects together when both Li and H are adsorbed on graphene is undoping of graphene resulting in the Fermi level shifting back closer to the Dirac point while the low-lying band due to the H atoms remains intact. When examining the band structures of the H-terminated graphene nanoribbon and comparing the band structure with and without Li (Fig. S19), we find that not only does the Fermi level move higher, but also the degeneracy between two of the bands is lifted resulting in a gap near the Fermi level.

**Table S1** Extracted electrolyte resistance ($R_e$), charge-transfer resistance ($R_{ct}$), and solid electrolyte interphases resistance ($R_{SEI}$) of various selected graphene samples. All the impedance measurements were conducted after 30 charge/discharge cycles of each sample.

| | $R_e$ (ohm) | $R_{ct}$ (ohm) | $R_{SEI}$ (ohm) | $Q_{dl}$ ( $10^{-5}$ F $s^{(a-1)}$) |
|---|---|---|---|---|
| **GNF-1050C** | **4.699** | **351.3** | **73.95** | **2.19** |
| **GNF-1600C** | **7.15** | **179.7** | **259.1** | **0.92** |
| **GNF-1050C-H** | **5.725** | **28.75** | **108.6** | **1.94** |
| **GNF-1050C-H2** | **9.79** | **136.3** | **44.63** | **2.19** |

**Table S2** Raman measurement parameters used in this work.

| **Laser wavelength (nm)** | **Laser source** | **Spot size (mm)** | **Spectral resolution ($cm^{-1}$)** | **Laser power (mW)** | **Integration time (s)** |
|---|---|---|---|---|---|
| 473 | DPSS | 1.9 | 11-19 | 1.11 | 120 |
| 532 | DPSS | 1 | ~13 | 1 | 120 |
| 632.8 | HeNe | 2.5 | 15-26 | 2.02 | 120 |

**Table S3** Contents of existing species at β during the reaction.

| **Species** | **# of moles** |
|---|---|
| $C$ | $n_C^0$ |
| $H$ | $\beta$ |
| $H_2^*(g)$ | $n_{H_2^*}^0 - (1/2)\cdot\beta$ |

**Figure S1 | Scanning electron micrograph** (SEM) of three representative graphene samples used for this study: (a) GNF-1050C, (b) GNF-1050C-H, and (c) GNF-1600C.

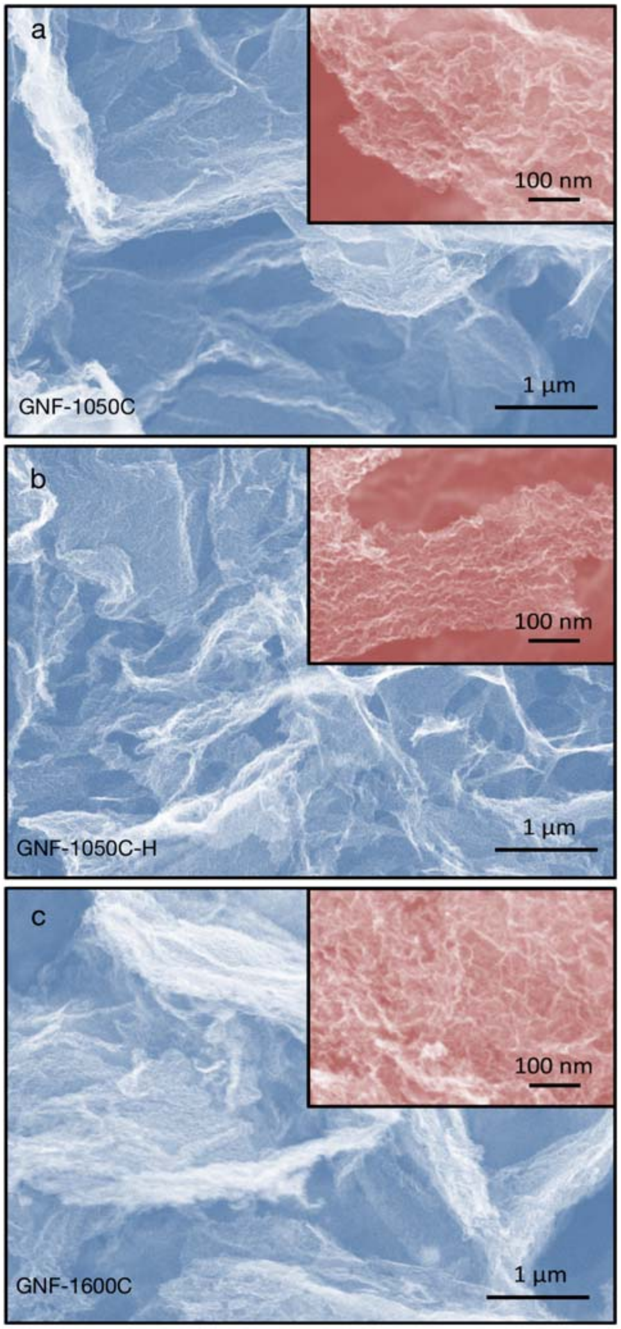

**Figure S2 | Energy dispersive D-band frequency at different laser excitation energies** for GNF-1050C and GNF-1050C-H samples.

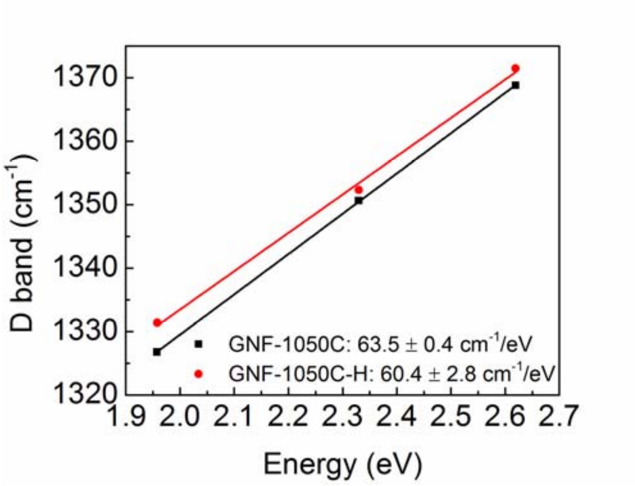

**Figure S3 | Calculated crystallite domain size for three graphene samples based on equation 3.** Our previous work (Carbon 85, 269-278, 2015) suggests that ion-beam introduced defects caused I(D)/I(G) ratio increase for GNF-1600C, suggesting that this material belongs to Stage I. In contrast, more defective GNF-1050C and GNF-1050C-H samples have a lower I(D)/I(G) ratio, suggesting that both these two materials belong to Stage II with much smaller crystallite domain size.

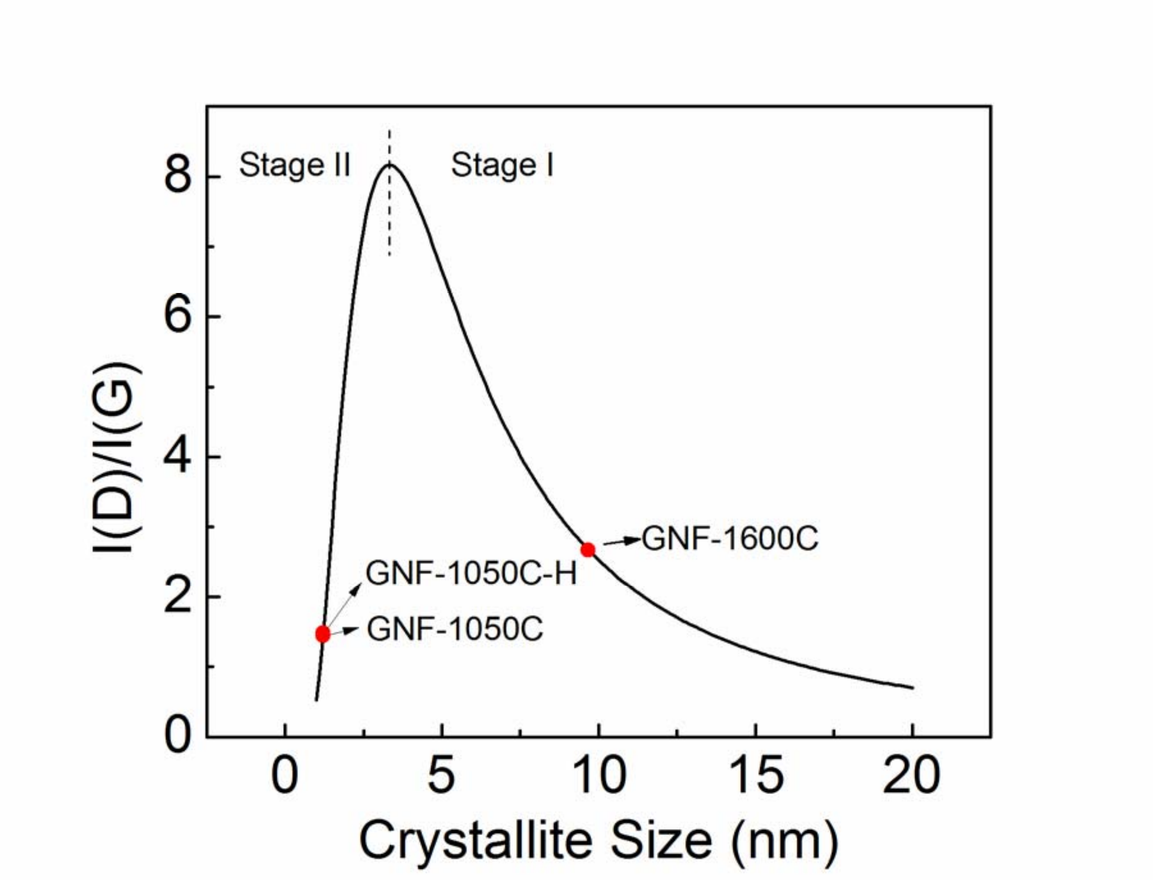

**Figure S4 | Pore size and pore volume**. (a) Nitrogen adsorption/desorption isotherm and (b) calculated pore volume and diameter distribution of three graphene samples.

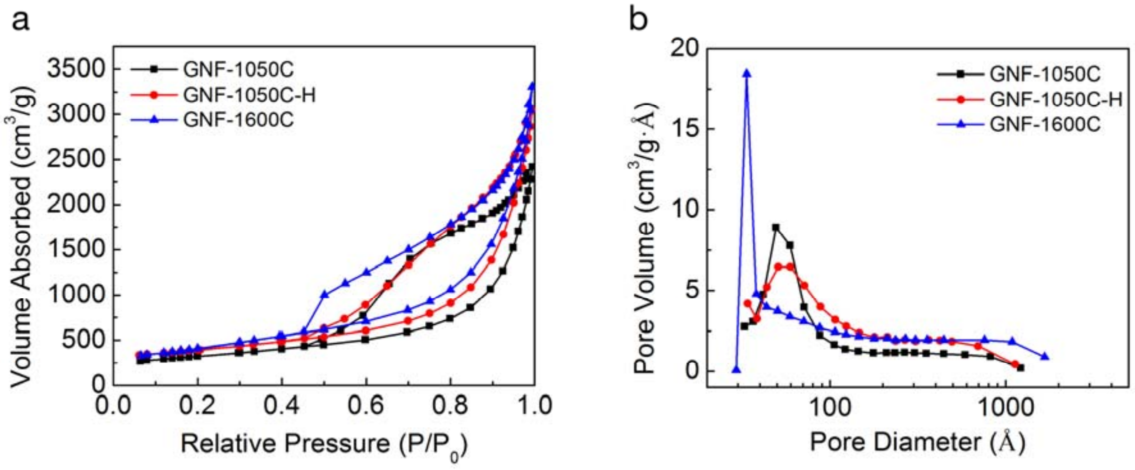

**Figure S5 | X-ray diffractometry (XRD).** By fitting (002) XRD peaks, we estimate average d-spacings of 0.350 nm to 0.351 nm for of GNF-1050C and GNF-1050C-H samples, respectively. This suggests that the interlayer spacing change may not play a deterministic role in the electrochemical performance of these two materials.

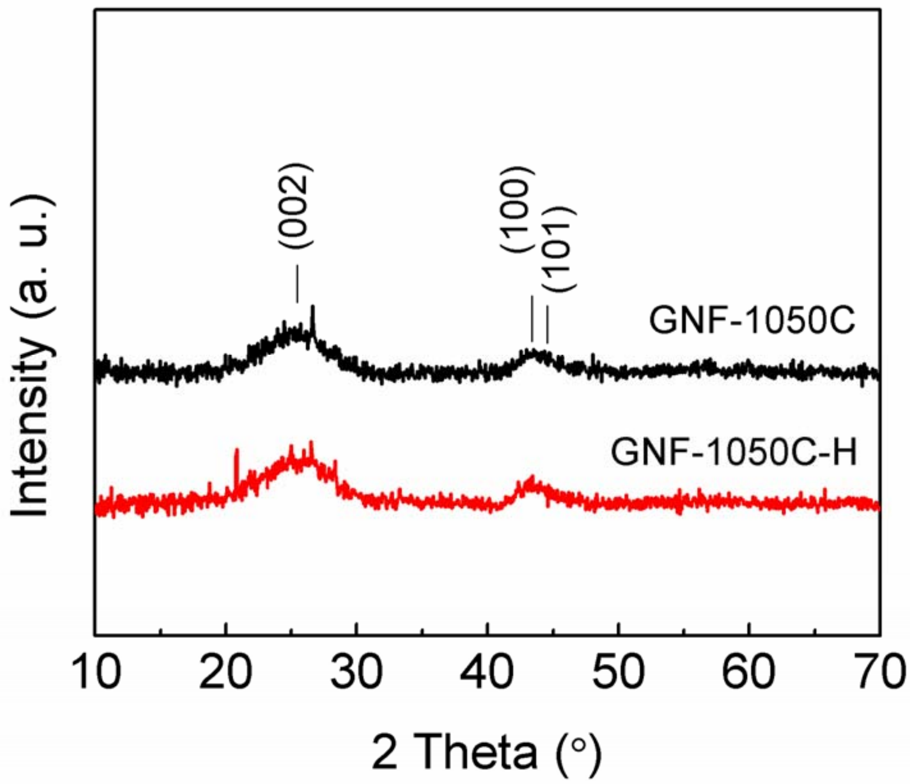

**Figure S6 | Electrochemical voltage profiles.** Charge/discharge voltage profiles of three representative graphene samples at (a) first, and (b) fifth cycle, respectively.

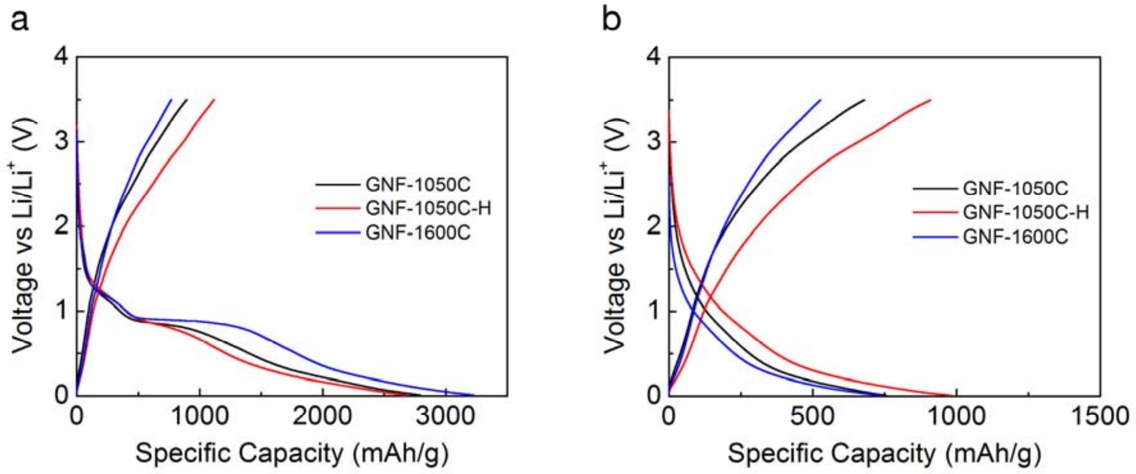

**Figure S7 | Rutherford backscattering spectrometry (RBS).** (a)-(d) RBS spectra of GNF-1050C and GNF-1050C-H, and GNF-1600 samples. Backscattered ion energies from O, N and C are indicated by arrows in RBS spectra. Insets in (a) and (b) are blowup images of the boxed region. The oxygen content remains approximately the same before and after $H_2$ treatment. GNF-1600C sample shows essentially zero oxygen content. (c)-(d) The nitrogen content measured in GNF-1050C and GNF-1050C-H is approximately the same (~1.8 at.%).

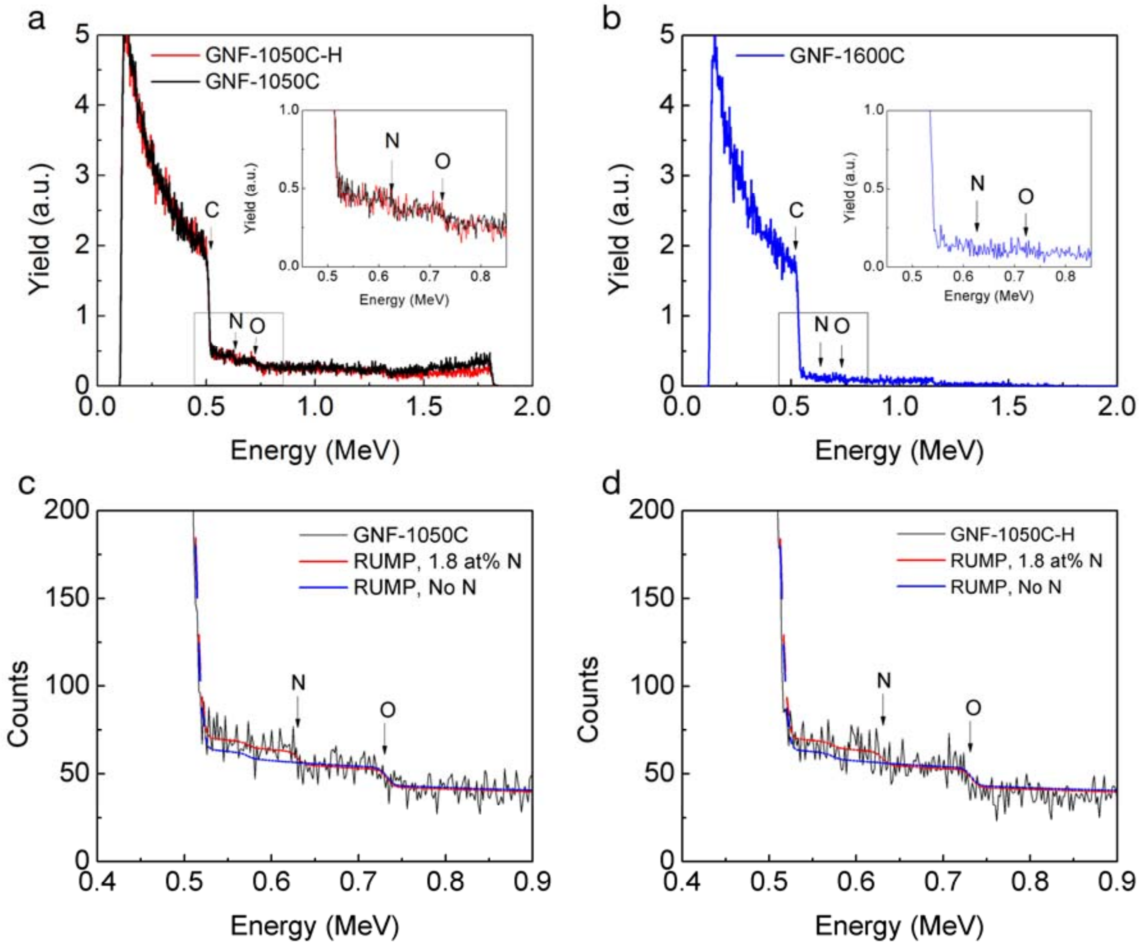

**Figure S8 | A comparison of electrochemical behaviour of GNF-1050C** (baseline sample), **GNF-1050C-H, GNF-1050C-H2** (annealed in 100% $H_2$ environment for 4h, 400 $^oC$), and **GNF-1600C**.

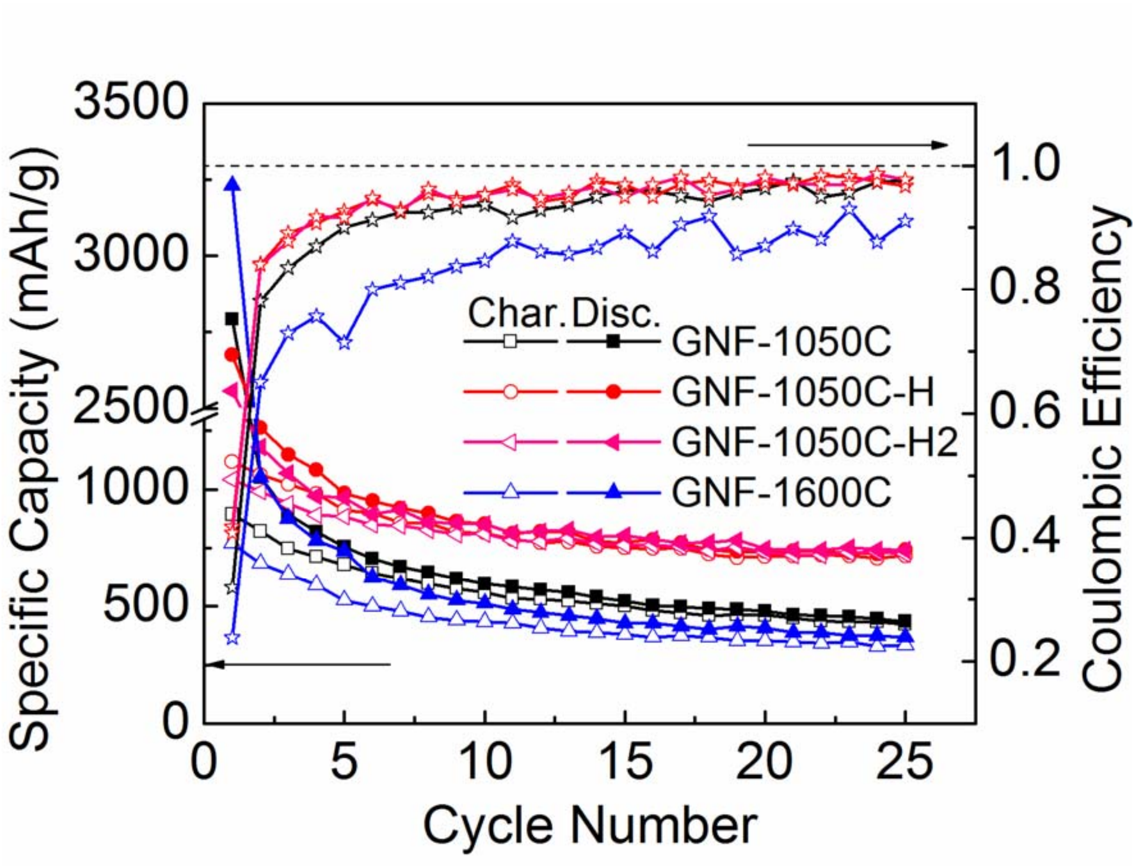

**Figure S9 | Electrochemical rate jump tests** for a series of GNF samples annealed at 1050 °C, 1600 °C, 2000 °C, and 2500 °C in Ar or He environment. Note the continuous reduction of specific capacity for samples annealed at higher temperatures.

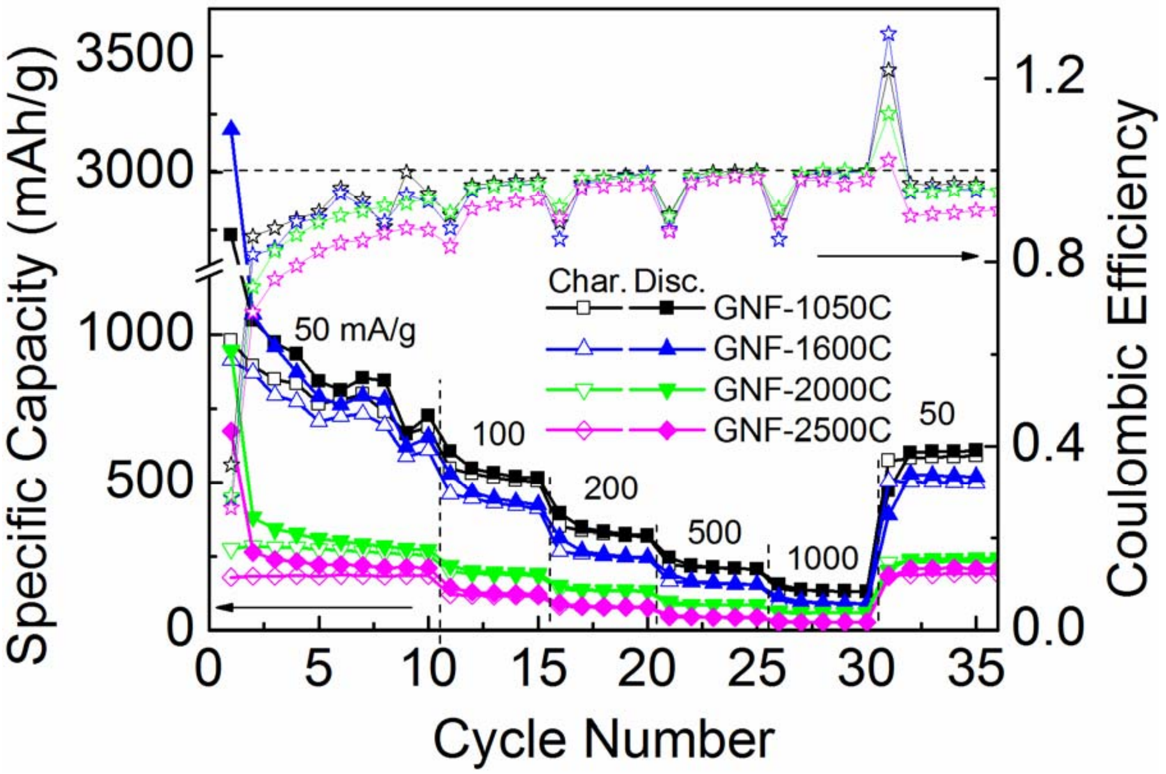

**Figure S10 | Schematic of Randles equivalent circuit model** used to calculate the resistance of various components in GNF electrodes.

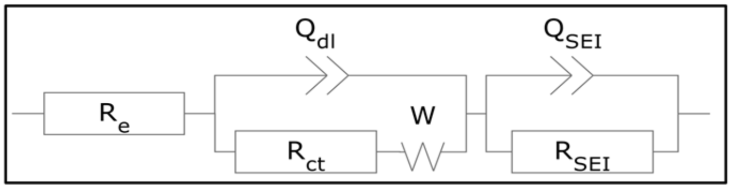

**Figure S11 | Electrochemical performance of GNF-1600C sample before and $H_2$ treatment (GNF-1600C-H)**.

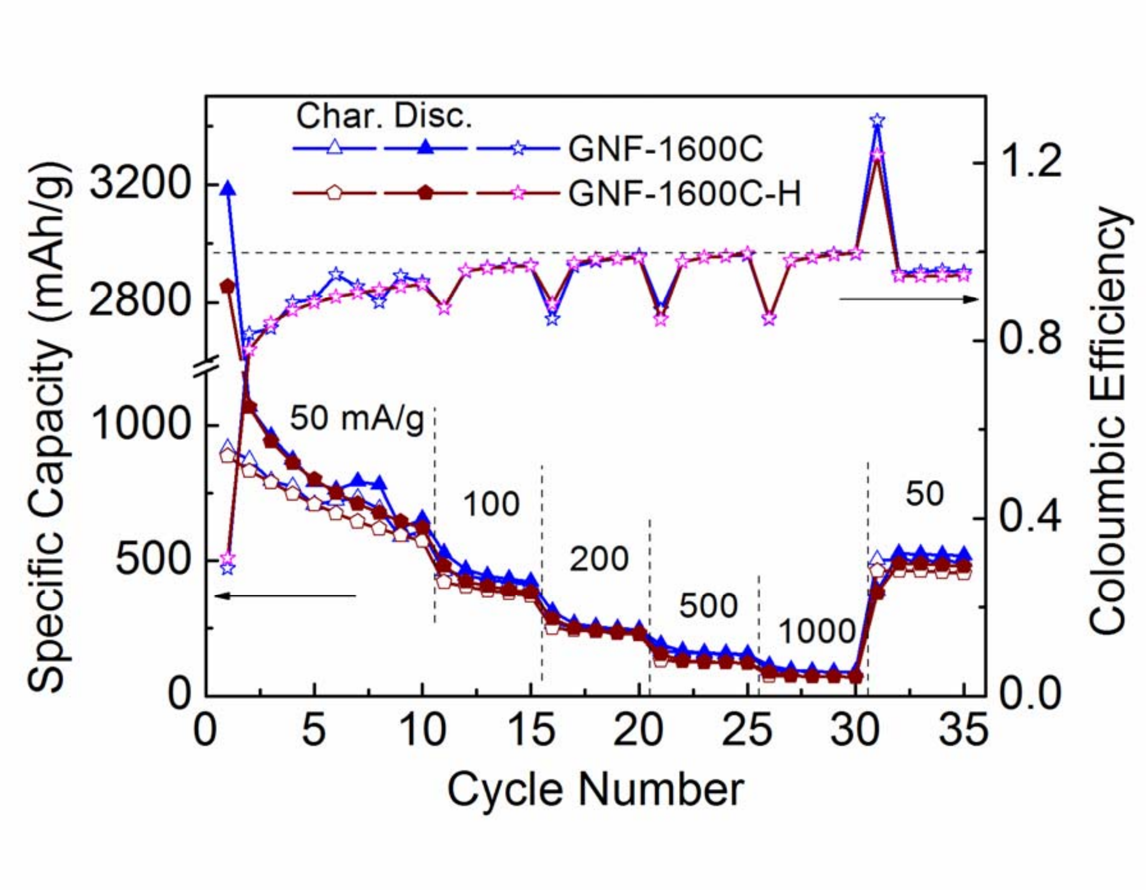

**Figure S12 | Schematic diagram of the experimental setup and conditions.** The same conditions will be applied in the thermodynamics modelling presented in Fig. 4a.

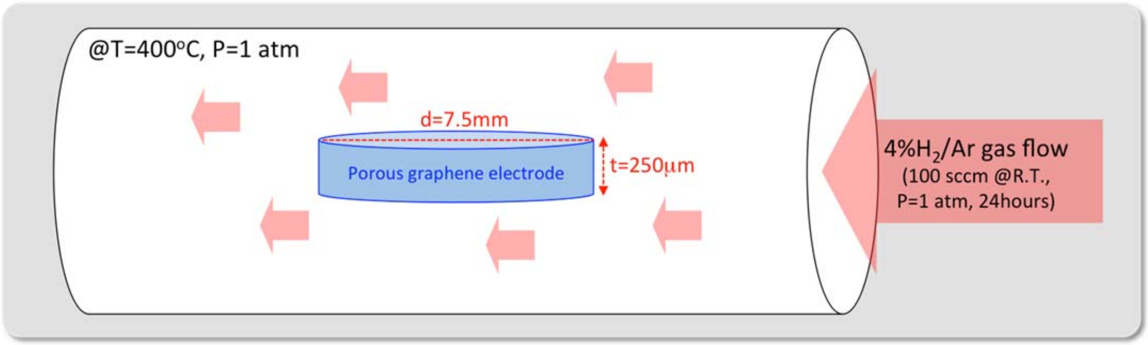

**Figure S13 | Estimated diffusion length of hydrogen on pristine graphene after 24 hours.** Distances are given as a function of temperature assuming a barrier of 1.06 eV (blue). Because the barrier is only an estimate, values are also plotted for ±0.2 eV from the computed barrier (dashed lines).

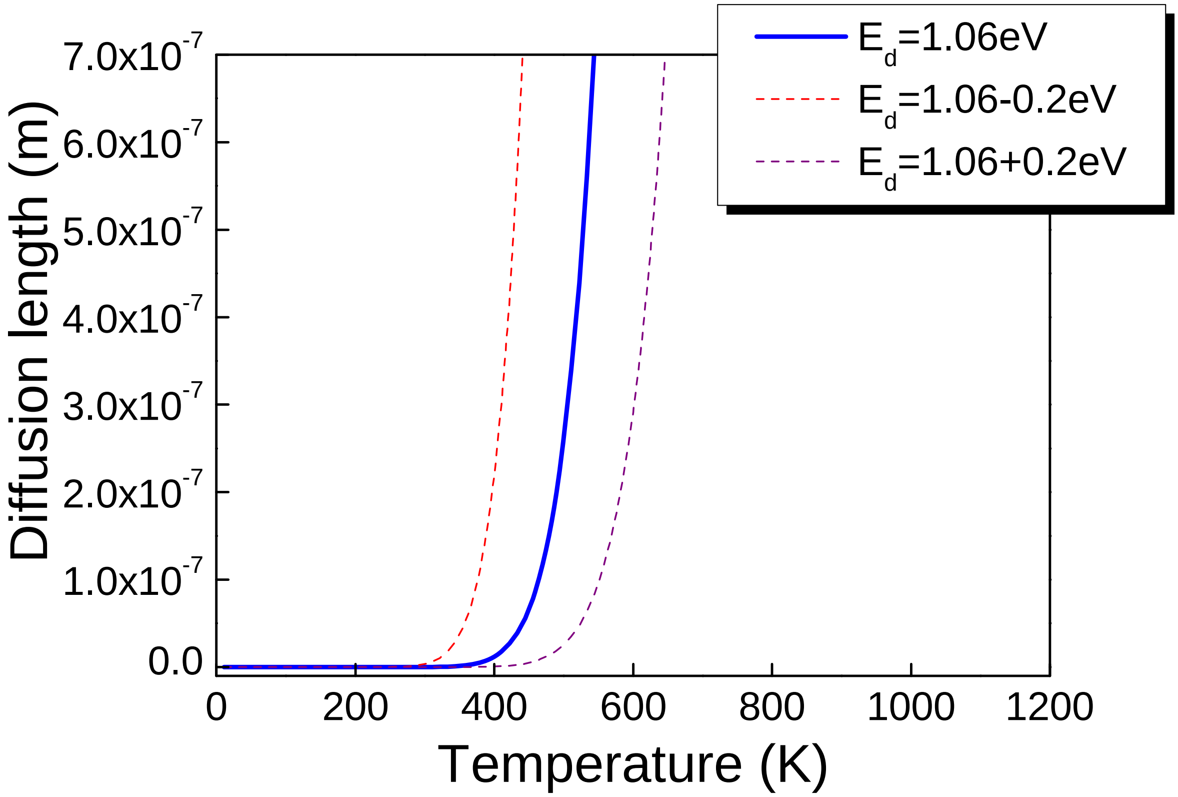

**Figure S14 | Effect of basal hydrogenation on interlayer spacing in bilayer graphene.** Hydrogen increases the interlayer spacing by 12%, facilitating $Li^+$ intercalation. Colour scheme: C=black, H=white, Li=green.

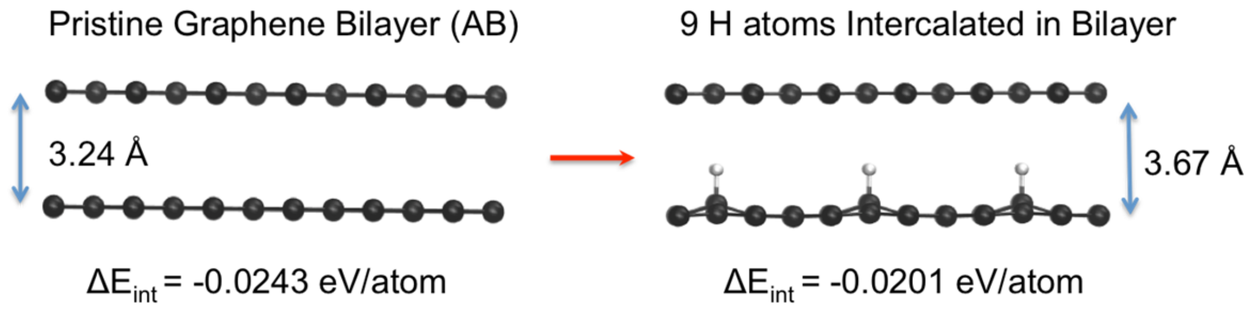

**Figure S15 | Enhanced Li binding on top and bottom of basal-H graphene.** Colour scheme: C=black, H=white, Li=green.

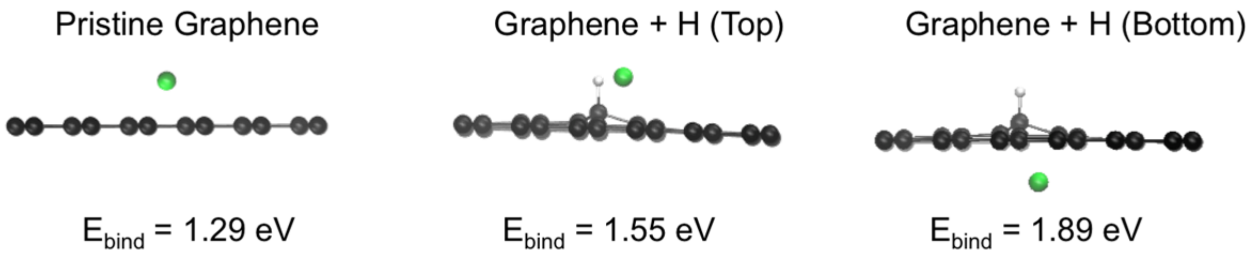

**Figure S16 | Enhanced Li binding near H-terminated edges of a graphene nanoribbon.** Both the initial (1$^{st}$) and subsequent (2$^{nd}$) differential binding energies are shown. Colour scheme: C=black, H=white, Li=green.

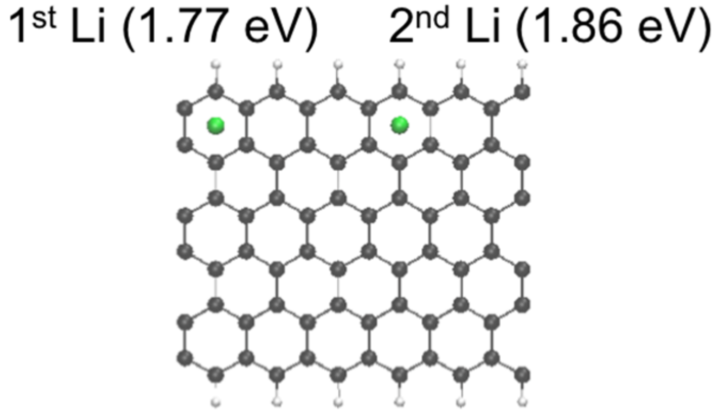

**Figure S17 | Binding of Li in the presence of H bridges.** Ortho, meta, and para bridge configurations are tested; only the meta configuration shows enhanced binding with respect to pristine graphene. The red arrow indicates the migration of the Li adsorbate from its original site during the atomic relaxation process. Colour scheme: C=black, H=white, Li=green.

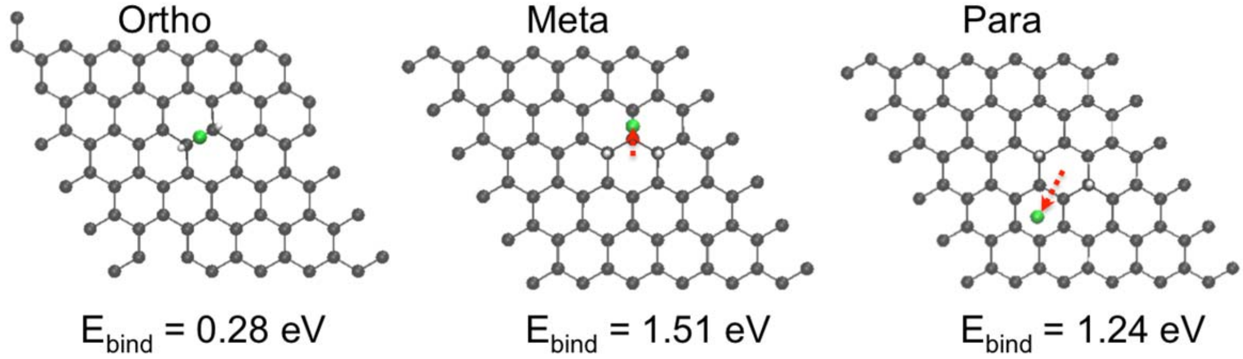

**Figure S18 | Electronic band structure of Li and H on the graphene basal plane.** Left to right: (i) pristine graphene; (ii) graphene with hydrogen; (iii) pristine graphene with adsorbed Li; (iv) graphene with hydrogen and adsorbed Li. Energies are referenced to the Fermi level.

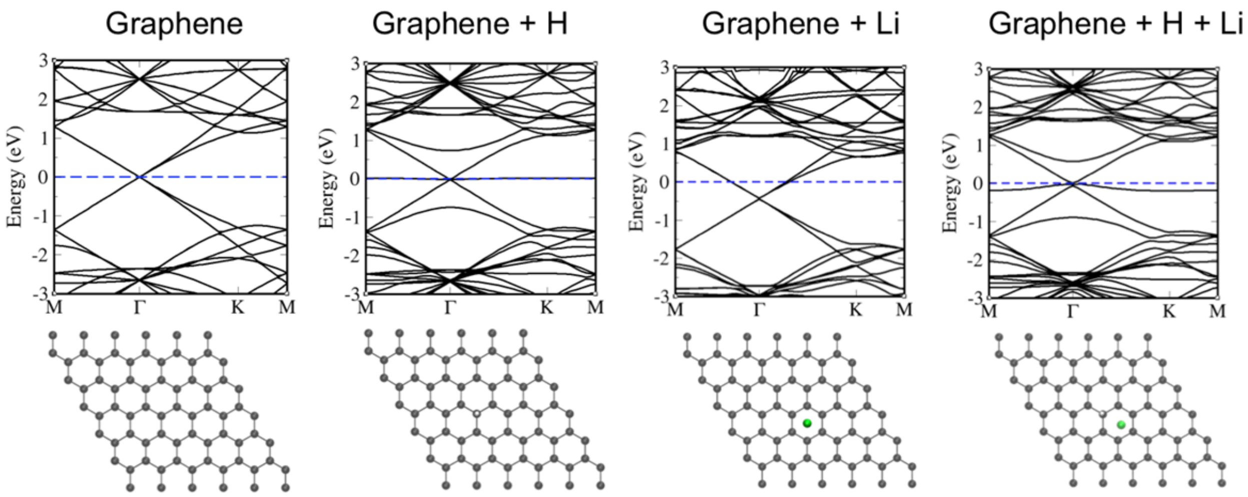

**Figure S19 | Electronic band structure of Li and H at graphene nanoribbon edge.** Left to right: (i) pristine graphene sheet; (ii) graphene nanoribbon with hydrogen termination; (iii) graphene nanoribbon with hydrogen termination and Li adsorbed at the near-edge site. Energies are referenced to the Fermi level.

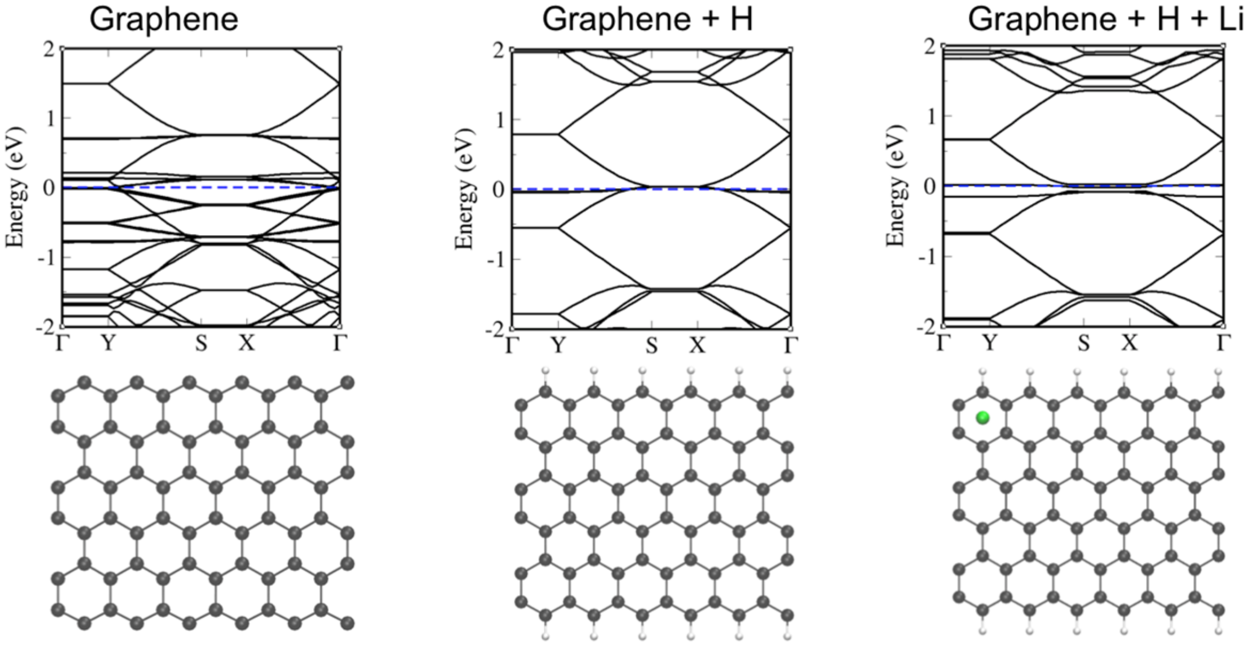

Supplement: Supplementary Information [file srep16190-s1.pdf]
